# Green Synthesized sAuNPs as a Potential Delivery Platform for Cytotoxic Alkaloids

**DOI:** 10.3390/ma16031319

**Published:** 2023-02-03

**Authors:** Byron Mubaiwa, Mookho S. Lerata, Nicole R. S. Sibuyi, Mervin Meyer, Toufiek Samaai, John J. Bolton, Edith M. Antunes, Denzil R. Beukes

**Affiliations:** 1 School of Pharmacy, University of the Western Cape, Robert Sobukwe Road, Bellville 7535, South Africa; 2 Department of Science and Innovation/Mintek Nanotechnology Innovation Centre (DST/Mintek NIC), Bio-Labels Node, Department of Biotechnology, University of the Western Cape, Robert Sobukwe Road, Bellville 7535, South Africa; 3 Department of Environmental Affairs (Oceans and Coasts), Cape Town 8000, South Africa; 4 Department of Biodiversity and Conservation Biology, University of the Western Cape, Robert Sobukwe Road, Bellville 7535, South Africa; 5 Department of Biological Sciences, University of Cape Town, Rondebosch 7701, South Africa; 6 Department of Chemistry, University of the Western Cape, Robert Sobukwe Road, Bellville 7535, South Africa

**Keywords:** pyrroloiminoquinones, gold nanoparticles, *Sargassum incisifolium*, Latrunculid sponges, *Tsitsikamma favus*, cytotoxicity

## Abstract

The use of natural products as chemotherapeutic agents is well established. However, many are associated with undesirable side effects, including high toxicity and instability. Previous reports on the cytotoxic activity of pyrroloiminoquinones isolated from Latrunculid sponges against cancer cell lines revealed extraordinary activity at IC_50_ of 77nM for discorhabdins. Their general lack of selectivity against the cancer and normal cell lines, however, precludes further development. In this study, extraction of a South African Latrunculid sponge produced three known pyrroloiminoquinone metabolites (14-bromodiscorhabdin C (**5**), Tsitsikammamine A (**6**) and B (**7**)). The assignment of the structures was established using standard 1D and 2D NMR experiments. To mitigate the lack of selectivity, the compounds were loaded onto gold nanoparticles synthesized using the aqueous extract of a brown seaweed, *Sargassum incisifolium* (sAuNPs). The cytotoxicity of the metabolites alone, and their sAuNP conjugates, were evaluated together with the known anticancer agent doxorubicin and its AuNP conjugate. The compound-AuNP conjugates retained their strong cytotoxic activity against the MCF-7 cell line, with >90% of the pyrroloiminoquinone-loaded AuNPs penetrating the cell membrane. Loading cytotoxic natural products onto AuNPs provides an avenue in overcoming some issues hampering the development of new anticancer drugs.

## 1. Introduction

Cytotoxic natural products such as vincristine, vinblastine, paclitaxel, doxorubicin, podophyllotoxin, camptothecin, and ecteinascidin-743 have been the mainstay of cancer chemotherapy for decades [1]. However, one of the major drawbacks of these drugs is their non-selective action in killing both cancer and normal cells [2,3]. In recent years, several nano-delivery systems have been developed for anticancer drugs in the hope of improving selectivity and thus reducing toxicity [4].

Of particular interest to this study was nano-delivery systems based on gold nanoparticles (AuNPs). The advantages of the AuNP-based delivery platforms include: (1) their biocompatibility, (2) ability to passively target tumors through the leaky vasculature (enhanced permeability and retention effect), (3) controlled release in response to internal or external stimuli, and (4) the ability to modify the AuNPs surface with targeting ligands to enhance tumor selective accumulation compared to free drugs [5]. Several studies have demonstrated the effective delivery and/or improved activity of several anticancer drugs using this approach. Two main strategies have been developed to load the AuNP with the anticancer drug. Firstly, through covalent linking via thiolated derivatives, and secondly, by loading the drug onto polysaccharide-capped AuNPs. These studies have demonstrated more selective targeting [6], intracellular delivery of the drug [7], in vivo stability and efficacy [8,9], lower plasma levels of the free drug (cf. AuNP bound) and reduced general toxicities in normal tissues [9].

Biological materials employed in synthesizing nanoparticles possess excellent reducing (e.g., polyphenols) and stabilizing agents (e.g., polysaccharides) [10]. The main advantage of loading the anticancer drug onto polysaccharide-capped AuNPs is the simple, green synthetic methods employed using biodegradable polymers compared to the more complex synthetic steps, and the toxic coupling reagents currently used to covalently link the anticancer drug to the AuNP [10]. Several different types of polysaccharide-based capping agents have been used to deliver anticancer drugs, including fucoidan [11], carboxymethyl xanthan gum [12], carrageenan [13] and pectin [14]. These studies also demonstrated improved cytotoxic activities in vitro, high drug-loading and good stability under varying pH and electrolytic conditions as well as pH-triggered drug release.

**Figure 1:**
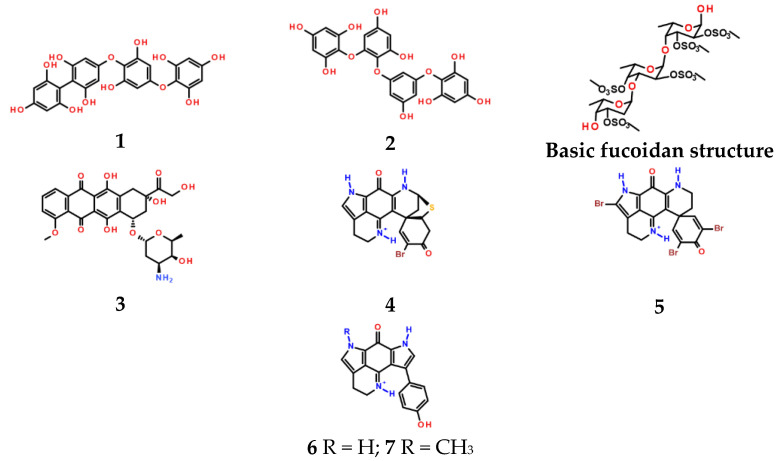
Structures of the compounds referred to or used in this study: fucodiphlorethol (**1**), tetraphlorethol (**2**), Doxorubicin (**3**), Discorhabdin A (**4**), 14-bromodiscorhabdin C (**5**), Tsitsikammamine A (**6**) and Tsitsikammamine B (**7**).

We have previously reported on the excellent redox properties of *Sargassum incisifolium* extracts [15] and demonstrated the simple, room temperature synthesis and characterization (FTIR, TEM, XRD etc.) of AuNPs (sAuNPs) using aqueous extracts of *S. incisifolium* (SiAE) [16]. The brown seaweed, *S. incisifolium* is widely distributed along the South African coastline from Cape Point to Mozambique [17]. Metabolites isolated from *S. incisifolium* organic extracts, such as fucoxanthin and sargaquinal, have demonstrated promising antimalarial bioactivities against chloroquine-sensitive strains of *P. falciparum* [18]. The aqueous extracts of *S. incisifolium*, however, is rich in antioxidant molecules which are responsible for the reduction of Au^3+^ to Au^0^ (AuNPs), while the water-soluble polysaccharides serve as capping agents [10,16]. Phlorotannins, e.g., fucodiphlorethol (**1**) and tetraphlorethol (**2**), are the main antioxidants in marine brown algae and are complex polymers of phloroglucinol (1,3,5-trihydroxybenzene) (Figure 1). The main water-soluble polysaccharides in the *Sargassum* spp. are alginic acid, fucoidan and laminaran. Alginic acid is a carboxyl-containing polysaccharide formed by joining β-D-mannuronic acid and α-L-guluronic acid through β-(1→4)/α-(1→4) glycosidic bonds. Fucoidan (Figure 1) is a water-soluble sulphated heteropolysaccharide with L-fucose groups linked through α-(1→3) or (1→4) glycosidic bonds [19], while laminaran is mainly composed of β-D-glucose bonded through β-(1→3) glycosidic bond links [20,21] (Figure 1).

The selective delivery and anticancer effects of AuNPs loaded with anticancer drugs can only be confirmed by in vivo studies. However, in this preliminary study we investigated the synthesis, characterization, and preliminary cytotoxic activity of *S. incisifolium* synthesized AuNPs (sAuNPs) loaded with doxorubicin (**3**) and the pyrroloiminoquinone alkaloids 14-bromodiscorhabdin C (**5**), and tsitsikammamine A (**6**) and B (**7**). If the AuNP-drug conjugate has the same, or improved activity, against a cancer cell line in vitro, then it may also demonstrate reduced toxic side effects due to the selective delivery of the conjugates to the cancer tissues. The pyrroloiminoquinones isolated from Latrunculid sponges have shown potent but non-selective cytotoxicity against both normal and cancer cell lines, which precluded their further development as anti-tumor drugs [22]. The targeted delivery, and increased selectivity, of these pyrroloiminoquinone alkaloids to tumor cells using nanoparticles as a delivery vehicle presents a highly attractive endeavor. In addition, the development of advanced targeted drug delivery system platforms for doxorubicin also remains a worthwhile research effort since an ideal, targeted, doxorubicin delivery platform decreases the required concentrations needed as well as the prevalence and intensity of side effects associated with the drug, while still utilizing its potent anti-cancer properties. In this work, we intend to show that (a) the sAuNPs are biocompatible and highly stable under physiological conditions, (b) that conjugation of the sAuNPs with doxorubicin (**3**) and the pyrroloiminoquinones (**5**–**7**) can be achieved, (c) that the ‘drug’ payload is released, and (d) that the AuNPs bound to cytotoxic alkaloids are able to traverse the MCF-7 cell membrane to exert their cytotoxic activity.

## 2. Materials and Methods

**Scheme 1:**
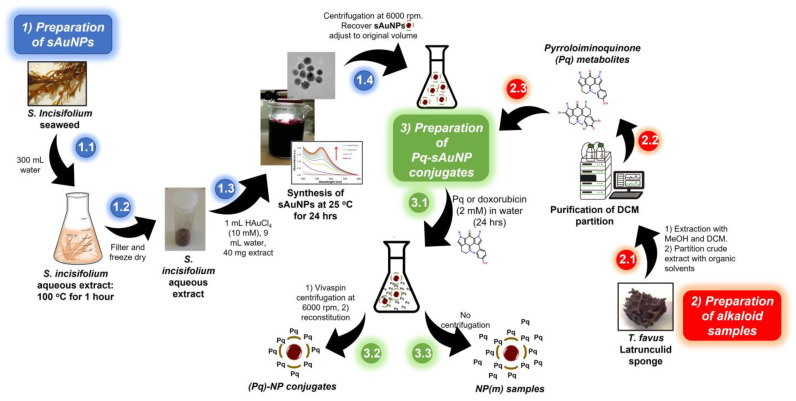
Preparation of the (1) *S. incisifolium* aqueous extract capped AuNPs (sAuNPs), (2) purification of the pyrroloiminoquinone (Pq) alkaloid metabolites and (3) sAuNP conjugates.

This section, as shown in Scheme 1, describes the preparation of the aqueous extracts of *S. incisifolium* (steps 1.1 and 1.2), the synthesis of sAuNPs (steps 1.3 and 1.4), the extraction and purification (steps 2.1 and 2.2) of the pyrroloiminoquinone (Pq) alkaloids from *T. favus* latrunculid sponge, and the preparation of the sAuNP conjugates (Pq-sAuNPs, step 3.1) with the pyrroloiminoquinone metabolites (and doxorubicin) using centrifugation (step 3.2, (Pq)-NP) or no centrifugation (step 3.3, NP(m)).

### 2.1. General Experimental/Materials

Gold(III) chloride (HAuCl_4_.3H_2_O), sodium citrate, Sartorius^®^ Vivaspin™ Centrifugal Concentrators (Polyethersulfone (PES) membrane, MWCO = 30 kDa), and Dialysis sacks were obtained from Merck-Sigma-Aldrich (St. Louis, MO, USA). The solid phase extractions (SPE) of the Latrunculid sponge were carried out on pre-packed Phenomenex^®^ Sep-Pak^®^ C18 cartridges.

Semi-preparative high performance liquid chromatography (HPLC) was carried out using an Agilent Technologies HPLC equipped with a Phenomenex^®^ Luna C18 (2), 10 µm (250 × 10 mm i.d.), a refractive index detector at 35 °C as well as a variable wavelength (at 245 nm) detector. NMR samples were prepared in deuterated solvents, and all experiments were acquired on a Bruker Avance III HD 400 MHz spectrometer equipped with a 5 mm BBO probe at 298 K using standard 1D and 2D pulse programs. Chemical shifts were referenced to residual undeuterated solvent signals in deuterated solvents peaks (DMSO-*d*_6_: δ_H_ 2.5, δ_C_ 39.51; CD_3_OD: δ_H_ 3.31, δ_C_ 49.15; D_2_O: δ_H_ 4.70) and reported in ppm. DOSY experiments were carried out using the 2D Bruker DOSY bipolar pulse program with longitudinal eddy current delay, ledbpgp2s at 298 K using a gas flow rate of 400 lph without sample spinning. The diffusion time δ was kept at 100 ms, while the pulse field gradient (δ/2) was adjusted to 2000 μs to acquire 2–5% residual signals with maximum gradient strength. The eddy current delay was 5 ms and the delay for gradient recovery was set to 0.2 ms. The gradient strength was incremented in 25 steps from 2% to 98% of its maximum value in a linear ramp. The data were processed using the Bruker TopSpin 3.6.2 software. An Agilent Technologies Cary-60 UV-Visible spectrophotometer was used for UV-Visible measurements using quartz cuvettes (10 mm). The size, shape, crystallinity, and elemental composition of the AuNPs produced were determined using TEM measurements, coupled with EDX and SAED detectors. The TEM images, EDX data and SAED patterns were obtained using a Tecnai F20 TEM with a field-emission gun (FEG) operating in bright field mode at 200 kV, using a lacy carbon mesh on top of a copper grid and dried under an electric bulb. ImageJ was used to measure the AuNP sizes in the TEM images. A Malvern^®^ Zetasizer Nano ZS™ was for all zeta potential and hydrodynamic particle size, while the powdered X-ray Diffraction patterns were acquired on a Bruker AXS (Germany) D8 Advance diffractometer (voltage 40 kV; current 40 mA). The XRD spectra were recorded in the range 30–90° using a CuKα (λ = 0.154 nm) monochromatic radiation X-ray source. Determination of the gold concentrations in the solutions was then accomplished using a Thermo ICap 6200 inductively coupled plasma optical emission spectroscopy (ICP-OES) utilizing a wavelength of 242.4 nm for Au at the Central Analytical Facility at Stellenbosch University. The instrument was calibrated and validated using NIST (National Institute of Standards and Technology, Gaithersburg, MD, USA) traceable standards to quantify the Au. Cell studies were performed under a class II biological safety cabinet. To visualize the cells, a Nikon light microscope with 20× magnification was used together with a Leica EC digital camera.

### 2.2. Extraction and Isolation of Pyrroloiminoquinones from Tsitsikamma Favus

The *Tsitsikamma favus* Latrunculid sponge, collected at the Tsitsikamma Marine Reserve, Eastern Cape, South Africa (GPS coordinates: −34.01515185009414, 23.81832453533402) and stored in a freezer at −20 °C, was collected (by SCUBA diving) and identified by Dr. Toufiek Samaai. A voucher specimen for the sample is housed in the School of Pharmacy, University of the Western Cape, South Africa. The mass of the sponge, after extraction and drying, was 240 g. The diced, thawed sponge was initially soaked in water (~2 L), the water decanted, and the sponge sequentially extracted with methanol (~2 L) and dichloromethane-methanol (1:1, ~2 L), for 48 h. The DCM-MeOH extract was dried and re-dissolved in MeOH and sequentially extracted with hexane, DCM and EtOAc giving a hexane fraction (Fr. A, 2.73 g), a DCM fraction (Fr. B, 269 mg), an EtOAc fraction (Fr. C, 101 mg) and an aqueous fraction (Fr. D, 620 mg). Fractions B, C and D revealed the characteristic ^1^H NMR signals for discorhabdins and tsitsikammamines and were fractionated therefore subjected to further isolation. Fractions B, C and D were further fractionated on C18 SPE cartridges using a step-gradient from 100% water to 100% MeOH. Final purification was achieved by repeated semi-preparative reversed-phase HPLC using a flow rate of 3 mL/min and 20% MeOH (0.05% TFA) as the eluent to give compounds **5**–**7**.

Compounds isolated:

14-Bromodiscorhabdin C (**5**): bright-red solid, isolated as a TFA salt; NMR data consistent with published data [23] and available in the supplementary document. HRESIMS *m*/*z* 539.8521 [M]^+^ (calcd. for C_18_H_13_N_3_^79^Br_3_O_2_, 540.8636). Yield: 15.5 mg.

Tsitsikammamine A (**6**): reddish-brown solid isolated as a TFA salt; NMR data consistent with published data [23] and is available in the supplementary document. LRESIMS *m*/*z* 304. 7 [M]^+^ (calcd. for C_18_H_14_N_3_O_2_, 304.1086). Yield: 9.0 mg.

Tsitsikammamine B (**7**): dark red solid, isolated as a TFA salt; NMR data consistent with published data [23] and available in the supplementary document. HRESIMS *m*/*z* 318.1237 [M]^+^ (calcd. for C_19_H_16_N_3_O_2_, 318.1242). Yield: 23.2 mg.

### 2.3. Preparation of Sargassum Incisifolium Aqueous Extracts (SiAE)

*Sargassum incisifolium* C Agardh, identified and collected by Prof. John Bolton, was attached on the intertidal rocky seashore at Noordhoek, Gqeberha (formerly Port Elizabeth), South Africa (GPS coordinates: −34.03967398674499, 25.63982817957248) and stored in a freezer at −20 °C. A voucher specimen is housed in the School of Pharmacy, University of the Western Cape, South Africa. The thawed seaweed was washed with Milli-Q water, frozen with liquid nitrogen and ground to a fine powder and freeze-dried. The powder (10.4 g) was extracted with methanol (200 mL) for 1 h at room temperature followed by extractions with dichloromethane-methanol (200 mL × 3) at 35 °C for 30 min. The extracted biomass was air-dried and finally extracted with water (300 mL, at 100 °C for 1 h). The extract was filtered and the filtrate freeze-dried to obtain a fine brown powder (SiAE) and stored at room temperature. The total phenolic content, reducing power and the radical scavenging power of the SiAE was determined by the methods described by Tobwala et al. [24] and Mmola et al. [16].

### 2.4. Synthesis of AuNPs

Systematic studies of the ratio of SiAE to HAuCl_4_ and reaction times led to the following optimized procedure: SiAE powder (20 mg) was added to HAuCl_4_ (1 mM, 10 mL). Aliquots (500 µL) were drawn from the reaction mixture and analyzed using UV-Visible spectroscopy every 30 s for 5 min and thereafter every 30 min for 5 h. Details on the optimization studies are included in the supplementary material.

Citrate capped AuNPs (cAuNPs) were also prepared according to well established literature methods [25] to enable comparison to sAuNPs.

### 2.5. Stability Studies

Measurements were acquired using a UV/Visible spectrometer scanning from 800 nm to 200 nm every 2.5 min. The effect of the NaCl concentration, pH, temperature, and freeze drying, interaction with HSA were all determined. The experimental details are given in the Supplementary Section.

### 2.6. Loading of Doxorubicin (***3***) and the Isolated Pyrroloiminoquinone Alkaloids (***5**–**7***) onto sAuNPs

Doxorubicin and the pyrroloiminoquinones (2.0 mM) were added to 2 mL of the prepared sAuNPs solution. This solution was divided into two portions. With the first portion, a 2 mL Vivaspin™ concentrator (MWCO =30 kDa) was used to filter off unbound pyrroloiminoquinone metabolites, trapping 200 μL of the pyrroloiminoquinone bound sAuNP conjugates (denoted **5**-NP, for example). The second portion was not applied to a Vivaspin concentrator, thus consisting of a mixture of bound and unbound pyrroloiminoquinones (denoted **5**-NP(m), for example). The preparation of the doxorubicin loaded NPs was similarly done, however, a drop of 0.1 M NaOH was added to assist in solubility of the doxorubicin conjugate and the mixture incubated for 24–48 h. A cellulose-based dialysis sack (MWCO = 12 kDa) was used to separate the unloaded or free doxorubicin from the sAuNP conjugates. The compound loaded sAuNPs were stored in the dark until required.

### 2.7. Drug Entrapment and Loading Efficiency

To determine the loading efficiency of doxorubicin, the unloaded drug concentration for the doxorubicin-sAuNPs was determined by UV/Visible spectroscopy at a wavelength of 290 nm and compared to a similar study carried out by Manivasagan et al. [11,26].

### 2.8. Biological Studies

A stock solution of 5 mg/mL of each alkaloid was prepared by incubating an appropriate quantity of alkaloid with the previously prepared sAuNP solution (Section 2.4) for 24 h (sAuNP-1). A portion of this incubated solution was centrifuged at 6000 rpm to remove unbound alkaloid and reconstituted to the original volume (sAuNP-2). These stock solutions were further diluted to give final concentrations in the range 1–50 μg/mL. The antiproliferative effects of the pure natural product, a mixture of natural product and sAuNP (sAuNP-1) and the natural product sAuNP mixture after removal of the unbound natural product by centrifugation (sAuNP-2) were assessed using the water-soluble tetrazolium salt (WST-1). MCF-7 cells were seeded in 96 well plates (100 µL of 1 × 10^4^ cells per well) and incubated for 24 h at 37 °C in a humidified incubator (maintained at 5% CO_2_). Fresh media containing 1–50 µg/mL of compounds **3**, **5**, **6** and **7** and their nanoconjugates, were added to their respective well and incubated for another 48 h. Post incubation, 50 µL of WST-1 dye was added to the cells and incubated for a further three hours. The absorbance in the wells were measured at a wavelength of 440 nm using a microplate reader. Cell viability (as a percentage) was then calculated according to Equation (1):

(1) $$\mathrm{Cell}\;\mathrm{viability}\;\left(\%\right)=\frac{\left(\mathrm{Absorbance}\;\mathrm{of}\;\mathrm{treated}\;\mathrm{cells}\right)}{\left(\mathrm{Absorbance}\;\mathrm{of}\;\mathrm{untreated}\;\mathrm{cells}\right)}\times 100$$

Average values for triplicates were used to calculate IC_50_ concentrations using QuestGraph™ IC_50_ Calculator: https://www.aatbio.com/tools/ic50-calculator (accessed on 8 January 2023).

### 2.9. Determination of sAuNP Uptake in MCF-7 Cells Using ICP-OES

sAuNP uptake was evaluated using ICP-OES. The MCF-7 cells were seeded in 12-well culture plates at a density of 1 × 10^5^ cells/mL (1 mL per well) and treated with sAuNPs-pyrroloiminoquinones/doxorubicin in triplicate for 48 h. The untreated samples were used as a negative control. Following treatment, the cells were washed twice with PBS. The cells were trypsinized and spun at 3000 rpm. The cell lysates were digested with 2 mL of *aqua regia* (HCl: HNO_3_, 3:1) at 90 °C for 2 h. The samples were then allowed to cool to room temperature. The volume of the digested material was adjusted to 10 mL with 2% HCl. The amount of Au taken up by the samples was analyzed using ICP-OES. The amount of AuNPs was calculated based on the concentration of Au found in each cell sample, and compared to the concentration used for treatment to obtain the percentage of AuNPs taken up by the cells (Equation (2)).

(2) $$\mathrm{Au}\;\mathrm{uptake}\;\left(\%\right)=\frac{{\left[\mathrm{Au}\right]}_{\mathrm{cell}}}{{\left[\mathrm{Au}\right]}_{\mathrm{total}}}\times 100$$

[Au]_cell_ is the Au content in the cell pellet and [Au]_total_ is the total Au used in the assay.

## 3. Results and Discussion

### 3.1. Characterization of the Pyrroloiminoquinone Alkaloids Isolated from Tsitsikamma Favus

The pyrroloiminoquinone natural products were extracted and purified from *T. favus* (Section 2.2) using C18 solid phase extraction and reversed phased HPLC. The structures of the natural products were confirmed by comparison of their one and two-dimensional NMR data with those reported in the literature [23]. Spectra and tabulated spectroscopic data can be found in the supplementary data.

### 3.2. Characterization of the Seaweed Aqueous Extracts

The aqueous extract of *S. incisifolium* (SiAE) containing polysaccharides and polyphenols, as the major and minor constituents, was prepared and analyzed using UV/Visible, IR as well as NMR spectroscopies, as previously reported [16]. The presence of the main constituents was confirmed using a multiplicity-edited HSQC NMR experiment (Figure S7). The HSQC data revealed a methyl signal at ~δ_C_ 18, the characteristic sugar oxymethine carbons between δ_C_ 60 and 80 and the anomeric carbons of the sugar moieties δ_C_ 100. The intensity of these signals together with the characteristic chemical shifts confirm the presence of a polysaccharide, namely the fucoidan, constituent. Additional, low intensity signals characteristic of phlorotannins/polyphenols were found at δ_C_/δ_H_ 102/6.2, suggesting that phlorotannins are also present, albeit as minor constituents in the *S. incisifolium* crude aqueous extract. The results obtained for the antioxidant and free radical scavenging assays were consistent with the findings obtained with Mmola et al. [16]. The authors reported the total phenolic content and total reducing power for the aqueous extracts to be 235 μg/mg GAE (Gallic Acid Equivalents) and 95 μg/mg AAE (Ascorbic Acid Equivalents) per mg of dried seaweed, respectively [16]. Diffusion ordered spectroscopy (DOSY) was also used to analyze the sample mixture by measuring the differences in the diffusion coefficient (Δ) of the molecules. The Δ value is related to the molecular weight, size, and shape of the molecule and is relevant to the surrounding environment, including temperature and solvent viscosity. The DOSY spectrum (Figure S8) obtained for the SiAE sample showed that the Δ values obtained were remarkably similar (Δ of 6.25 × 10^−10^ m^2^/s and 5.79 × 10^−10^ m^2^/s for the polyphenol and polysaccharide, respectively) indicating that both components in the mixture are large macromolecules. Water was observed with a Δ value of 2.32 × 10^−9^ m^2^/s.

### 3.3. Synthesis and Characterization of cAuNPs, sAuNPs and Drug Loaded AuNPs

**Figure 2:**
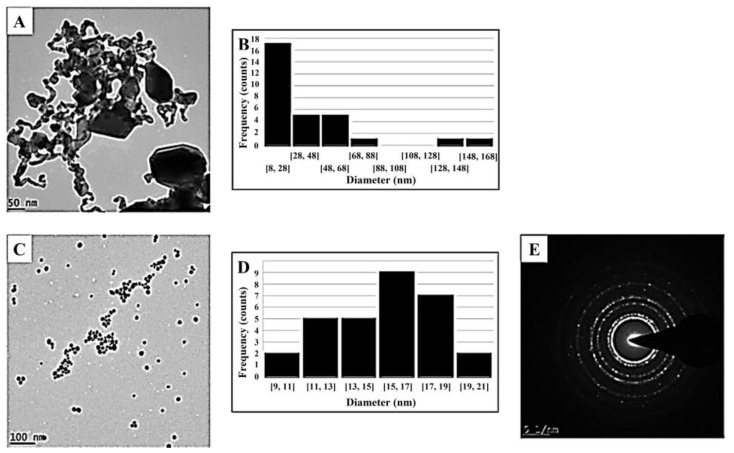
TEM images (**A**,**C**) and NP size distribution (**B**,**D**) for the cAuNPs prepared using a gold salt:citrate ratio of 2.5:1 (**A**,**B**) and the optimized ratio at 0.022:1 (**C**,**D**). The SAED (**E**) pattern obtained for the cAuNPs synthesized with a metal salt: citrate 0.022:1 ratio.

**Figure 3:**
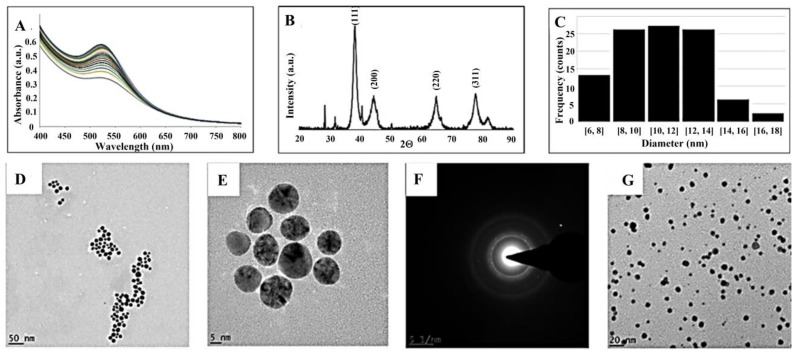
UV/Visible spectra (**A**) collected (in water) every 5 s for 24 h showing the sAuNP formation using SiAE, and the XRD powder pattern (**B**). The sAuNP size distribution (**C**) and TEM images (**D**,**E**), together with the SAED (**F**) pattern obtained for sAuNPs are also shown. The TEM image obtained for the sAuNPs following the processes of ultrafiltration/centrifugation and reconstitution is given in (**G**).

The rate of AuNP formation, stability, and morphology of the AuNPs synthesized were compared using citric acid as the control sample [25], and SiAE as the capping (and reducing) agents. The metal salt to citrate (cAuNPs) or metal salt to extract (sAuNPs) ratios were optimized and the stability of the optimal AuNPs produced for each method compared (Figure 2 and Figure 3 for cAuNPs and sAuNPs, respectively).

The citrate capped AuNPs formed only at higher temperatures (100 °C), and after 5 min an SPR band was observed at 524 nm. Due to the discrepancies in the literature with regards to the metal salt to capping ratio, the reaction conditions were optimized. The TEM images of the cAuNPs using a metal salt to citrate ratio at 2.5:1, revealed a wide range of nanoparticle shapes and sizes, including spherical and cuboid shapes ranging from 8 nm to 168 nm (Figure 2A,B), indicating that the metal salt concentration should be kept to a minimum. The cAuNPs formed at an optimized metal salt to citrate ratio of 0.022:1, revealed spherical, monodispersed NPs which were on average 14.5 nm in size (Figure 2C,D). The Selected Area Electron Diffraction (SAED) pattern revealed the cAuNPs to be crystalline (Figure 2E) in nature.

The sAuNPs were easily prepared at room temperature using the *S. incisifolium* aqueous extract (SiAE). UV/Visible absorption spectra revealed the characteristic surface plasmon resonance band at 525 nm (Figure 3A). The sAuNP formation proceeded at a faster rate with an increasing amount of extract (Figure S9), and optimal conditions were determined to be at a 0.167:1 ratio (metal salt: capping agent) for the sAuNPs. The TEM images for the sAuNPs, the size dispersion histogram and the SAED patterns are shown in Figure 3, together with the X-ray powder patterns. The TEM images (Figure 3D,E) showed spherically shaped, monodispersed sAuNPs which were 10.5 nm in size on average, with sizes ranging from 4 to 18 nm (Figure 3C). Inspection of the SAED pattern (Figure 3F) showed the sAuNPs to be polycrystalline, while the X-ray powder pattern (Figure 3B) confirmed the crystalline nature and face-centered cubic structure of the sAuNPs (2θ = 38.23° (111), 44.41° (200), 64.76° (220) and 77.77° (311)). Reflections due to NaCl and KCl were attributed to the presence of these salts in the seaweed. These data are consistent with that reported by Mmola et al. [16]. Dynamic light scattering (DLS) measurements, used to compliment the data obtained from the TEM analyses, helped to determine the hydrodynamic (Hd) diameter of the AuNPs suspended in solution and revealed information about the capping of the nanoparticles (Table 1). The DLS measurements for the sAuNPs (Table 1) revealed a hydrodynamic radius of 28.4 nm, a polydispersity index (PdI) of 0.242 and a zeta potential of −47.2 mV. The PdI indicates a broad size distribution, while the negative zeta potential indicated a negative surface charge, as expected for the polysaccharide capping on the NP surface [27]. The large negative charge is also associated with the inherent stability of the NP [28]. The cAuNPs, on the other hand, were found to be unstable and only the hydrodynamic radius (10.4 nm) was obtained.

### 3.4. Stability of the cAuNPs and sAuNPs

The stability of the synthesized sAuNPs were assessed against the standard citrate capped AuNP (control) for four weeks. The retention of the SPR band, the hydrodynamic radii as well as visual observations were recorded, and the stability of the NPs at various temperature and pH conditions were assessed (Table 1). The experimental methods are given in the Supplementary Materials.

As potential drug carriers, the AuNPs are expected to be stable and robust in the concentrations considered to be isotonic to blood plasma and red blood cells (to avoid lysis). Shukla et al. described a variety of biocompatible conditions requiring stability in the human body [28]. These include challenging the AuNPs at various NaCl concentrations (normal saline concentrations are isotonic at 154 mM), observing the interaction with human serum albumin (HSA) (one of the major plasma proteins involved in drug binding), temperature and pH [29]. Increasing the ionic strength (to 100 mM NaCl) resulted in a shift to longer wavelengths for the SPR band in the cAuNPs (Table 1 in parentheses) which is attributed to the aggregation of the cAuNPs. Changes in the zeta potential from −1.8 mV to more positive values, and the hydrodynamic diameters increasing to 811 nm (from 10.4 nm), all point to a decrease in the stability of the AuNP [30]. This contrasts sharply with the sAuNPs, which were found to be stable up to 5000 mM NaCl, i.e., 30 times the concentration of the medium isotonic. The sAuNPs retained their color and the SPR wavelength, with a slight increase in their hydrodynamic diameters observed. The ionic adsorption of Na^+^ and Cl^−^ ions are therefore not likely to destabilize the NP (see Figure S11). Observations (including UV/Visible spectra) revealed that the sAuNPs, were stable at low HSA concentrations. It is expected that the proteins will interact via electrostatic interactions, with the polysaccharide’s sulfate ions coating the sAuNP. Conversely, the cAuNPs were observed to immediately destabilize, coalesce, and flocculate. TEM images (Figure S12) revealed that the sAuNPs retained their structure, while the cAuNPs did not (Table 1). The sAuNPs also proved to be more stable at all temperatures and pH conditions, while the cAuNPs were not stable at low temperature and only stable at neutral pH (Table 1). The response of the sAuNPs to the various conditions was encouraging, as these conditions are related to the human body i.e., pH 7.4 (blood), pH 2 (stomach), pH 5.6–6.8 (intestines) and pH > 6.8 (tissues) [31].

### 3.5. Preparation of the Pyrroloiminoquinone (***5**–**7***) Loaded sAuNPs

**Figure 4:**
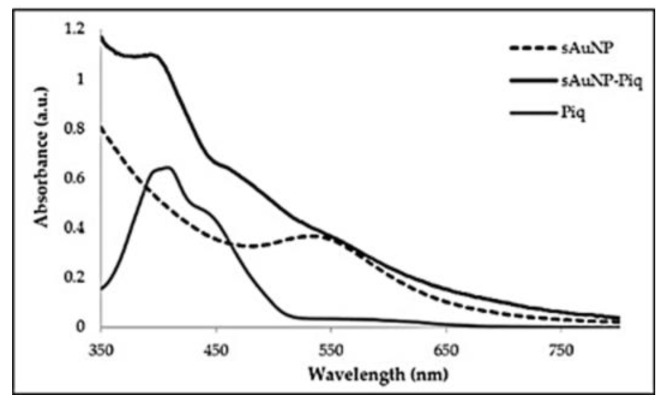
UV/Visible absorption spectra of the sAuNP, a representative pyrroloiminoquinone metabolite (BDC (**5**)), and their conjugate with the sAuNP in water.

The sAuNP-Pyrroloiminoquinone conjugates were prepared by loading the pyrroloiminoquinone metabolites (**5**, **6** and **7**) onto the sAuNPs as a DMSO solution (~2 mM). A 2 mL Vivaspin™ concentrator (MWCO: 30 kDa) filtered off unbound metabolites, while trapping 200 μL of the sAuNPs. The Vivaspin™ concentrator was used to separate the un-loaded or free pyrroloiminoquinones from the sAuNP-pyrroloiminoquinone conjugates, blocking molecules more than 5 nm in diameter. The cAuNPs turned blue and became insoluble when subjected to the same process. Representative UV/Visible spectra for the highly colored, unbound 14-bromodiscorhabdin C (**5**), the sAuNPs alone and the pyrroloiminoquinone loaded sAuNP are shown in Figure 4. The spectra for the conjugate reveal a typical profile which is the sum of the individual absorption spectra of the discorhabdin and the sAuNPs, as expected. No real shifts in the absorption maxima for the AuNP or the pyrroloiminoquinone were observed, and the materials are thus expected to be intact.

### 3.6. Preparation of the Doxorubicin (***3***) Loaded sAuNPs

**Figure 5:**
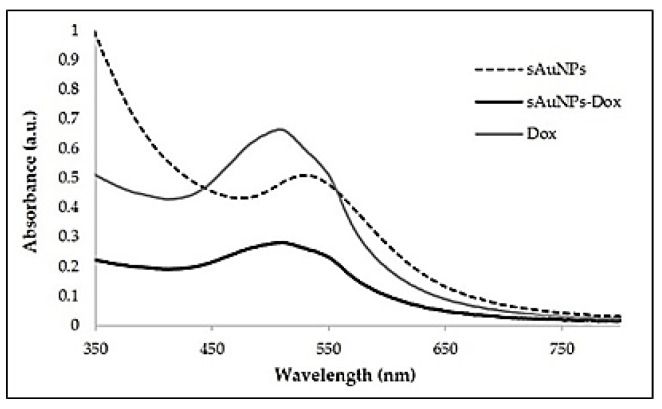
UV/Visible absorption spectra of doxorubicin alone, the sAuNPs and the sAuNP-doxorubicin (sAuNPs-Dox) conjugate in water.

**Figure 6:**
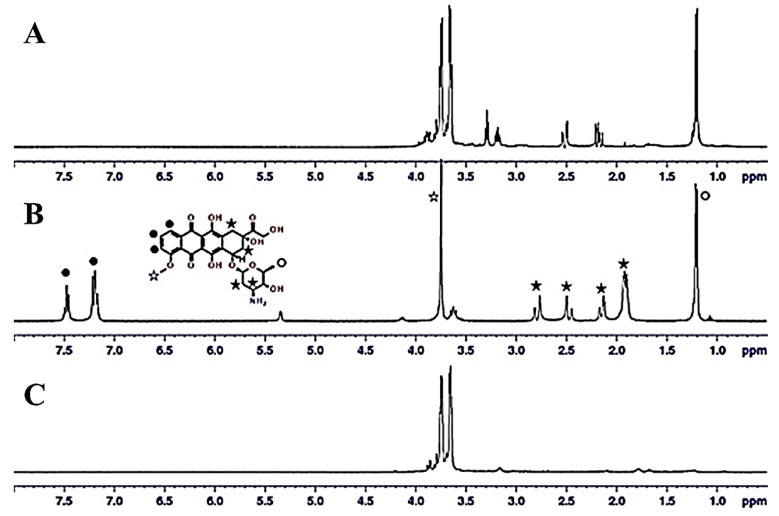
^1^H NMR spectra (400 MHz, D_2_O) of the sAuNP-doxorubicin conjugate (**A**), doxorubicin (**B**) and the sAuNPs alone (**C**) at 298 K using water suppression.

Loading of doxorubicin onto the sAuNPs required a few modifications, as adding doxorubicin as an aqueous solution (~2 mM) resulted in precipitation due to the acidic environment of the aqueous extract (sAuNPs have pH of ~4). A cellulose-based dialysis sack (MWCO: 12 kDa) was used to separate the unloaded or free compound from the sAuNP conjugates. A total of 20 mg of aqueous extract was used to produce the sAuNPs to enable comparison with a previously reported fucoidan-capped doxorubicin loaded AuNP [27], where the authors reported a loading as high as 90%. The UV/Visible absorption and ^1^H NMR spectra obtained for the sAuNPs-doxorubicin conjugate is shown in Figure 5 and Figure 6, respectively, together with the individual components. No obvious changes were observed for the conjugate upon comparison with the individual constituents.

The ^1^H NMR spectra obtained for doxorubicin, the sAuNPs and the conjugate are shown in Figure 6. The NPs alone (Figure 6C) show signals between δ_H_ 3.5 and 4 which are characteristic of polysaccharides, as expected.

Figure 6B shows the spectrum obtained for doxorubicin alone, with aromatic signals present between δ_H_ 7 and 7.5, an oxy-methyl moiety at 3.8, the diastereotopic proton signals of the two aliphatic rings between δ_H_ 1.8 and 2.8, and a methyl signal at δ_H_ 1.28. Interestingly, while the signals pertaining to the methyl at δ_H_ 1.28 and some of the diastereotopic protons between δ_H_ 1.8 and 2.8 remain upon conjugation, albeit with some significant changes in chemical shifts for the latter, the aromatic signals are noticeably absent. The shifts (Figure 6) in the methylene signals suggest a meaningful change in the ^1^H chemical environment for the aliphatic rings, perhaps through hydrogen bonding and various intermolecular forces, which is expected due to the polysaccharide capping on the AuNP surface. The absence of the aromatic protons suggests some binding to the gold nanoparticle surface through the aromatic rings, perhaps through cation -π stacking [32]. Curry et al. provided substantial practical and theoretical evidence, where the main absorption process for doxorubicin loading on citrate capped AuNPs were driven firstly through hydrophobic forces towards the AuNPs, followed by cation-π interaction primarily through coordination of the doxorubicin anthracene moiety to the cations on the AuNP (through diffusion-limited kinetics) [32]. The authors found that doxorubicin displaced the citrate capping agent easily, however, in this work, the ^1^H NMR signals due to fucoidan are still observed. This would thus confirm our observations for the absence of the doxorubicin aromatic proton signals. Curry et al. found that the contribution of the amine functional group to be negligible; this was confirmed through high resolution XPS measurements and fluorescence-based measurements [32]. The authors were also able to show that there was no evidence to support electrostatic interactions or salt-bridging between the positively charged amine moiety on doxorubicin, and the negatively charged citrate capping as the main mechanisms of doxorubicin loading. ^1^H NMR of the pyrroloiminoquinone-loaded-sAuNP conjugates were not obtained due to the paucity of material available.

### 3.7. Doxorubicin Release/Desorption

The release or desorption of doxorubicin from the doxorubicin loaded sAuNPs was also studied. Dialysis prevented the AuNPs from traversing the cellulose-based dialysis sack (MWCO: 12 kDa) but allowed the free movement of the unbound drug (i.e., the released doxorubicin). Periodic sampling up until 72 h was carried out and followed by UV/Visible absorption at 290 nm, which allowed the determination of the doxorubicin payload release as a function of time. The amount of doxorubicin released from sAuNPs-Dox at 20 °C and pH 7.4 after 72 h was determined to be 17%, as summarized in Table 2. This was comparable to the amount of doxorubicin released by fucoidan-capped AuNPs-doxorubicin (Dox-Fuc-AuNPs) reported by Manivasagan et al. [26].

### 3.8. sAuNP Cellular Uptake Analysis Using ICP-OES

**Figure 7:**
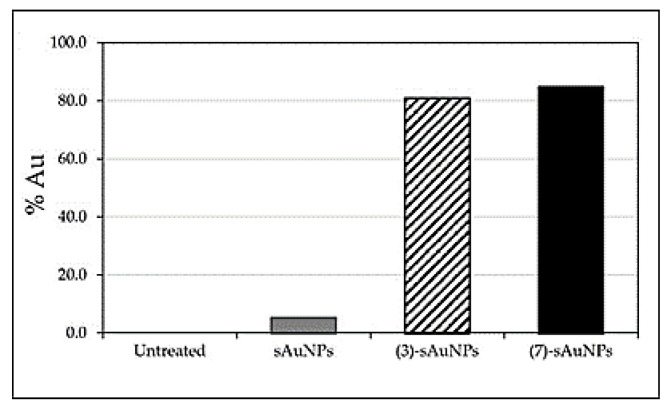
ICP-OES of cellular uptake of sAuNPs and its nanoconjugates by the MCF-7 cells.

It was not clear whether the AuNP-pyrroloiminoquinone conjugates (with its drug payload) could penetrate the MCF-7 cell membrane to exert its activity. ICP-OES was therefore chosen to determine the amount of gold present inside the MCF-7 cells. Following incubation and cell digestion of the nanoconjugates, centrifugation at low speeds (allowing the cells to sediment into pellets, but not the AuNPs themselves) enabled a comparison of the total amount of gold dosed to the cells, against the amount of gold found inside the cells. This permitted the estimation of the percentage penetration of the alkaloid loaded sAuNPs able to enter the cancer cell. Only the Tsitsikammamine B (**7**) and doxorubicin (**3**) sAuNP nanoconjugates were evaluated, and the results are presented in Figure 7.

The sAuNPs alone revealed poor uptake (5%) by the cancer cells (Figure 7), while the doxorubicin (**3**)- or pyrroloiminoquinone (**7**)-AuNP conjugates showed 80–85% penetration.

### 3.9. Biological Studies

**Figure 8:**
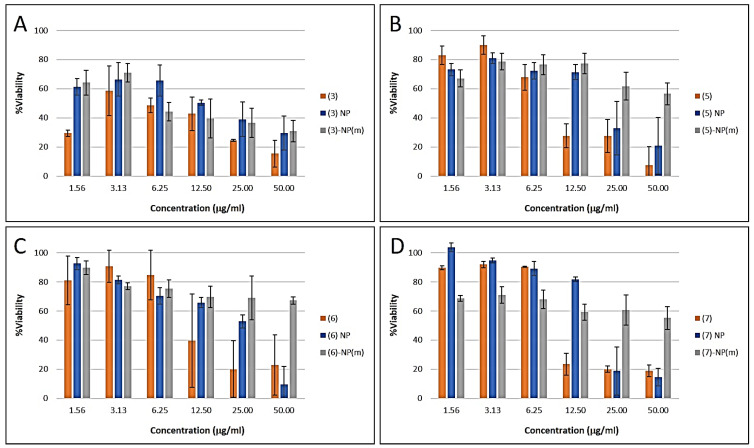
Cytotoxicity of doxorubicin (**3**), 14-bromo discorhabdin C (**5**) and the tsitsikammamines (**6** and **7**) alone, their as-synthesized sAuNP conjugates (NP) and the mixture (NP(m)) against the MCF-7 cancer cells. Dose: 1.5 μg/mL to 50 μg/mL. Graphs are labelled (**A**) doxorubicin (**3**) and conjugates, (**B**) 14-bromo discorhabdin C (**5**) and its conjugates, (**C**) Tsitsikammamine A (**6**) and its conjugates, (**D**) Tsitsikammamine B (**7**) and its conjugates.

Pyrroloiminoquinone metabolites have been previously shown to be highly cytotoxic, and were prospective candidates for development as anticancer drugs [21]. Discorhabdin A, the most cytotoxic pyrroloiminoquinone, revealed anti-tumor activity (IC_50_) in the range of 77 nM against the Human Colon Tumor cell line (HCT-116), while the cytotoxic data (IC_50_) obtained for compounds **5**, **6** and **7** were revealed to be 0.08, 1.4 and 2.4 μM, respectively. The role of the pyrroloiminoquinone structural motif plays in the high cytotoxicity was demonstrated by Antunes and co-workers (2004) though isolation and comparison of the relative activities of additional compounds such as Makaluvic acid A and Damirone B [22]. However, the cytotoxic activity of these compounds against cancer cell lines was also demonstrated in subsequent studies in normal cell lines, precluding their use in cancer treatment [22]. The possibility of selective targeting of cancer cells using pyrroloiminoquinone-loaded AuNPs as a carrier was therefore enticing. This could be achieved through selective uptake by cancer cells, or by selective delivery to tumor tissues via leaky vasculature. The antiproliferative activities of the pyrroloiminoquinone metabolites (**5**–**7**), doxorubicin (**3**) and their AuNPs conjugates (e.g., (**5**) NP) were assessed against the breast cancer (MCF-7) cancer cell line. In addition, the effect of any unbound drug was assessed by its removal through centrifugation. The pyrroloiminoquinone metabolites demonstrated dose dependent antiplroliferative activities in the range 1.5–50 μg/mL as shown in Figure 8 (Table 3, Figure S14).

The “unloaded” sAuNPs showed very low cytotoxicity, however, the pure natural products displayed IC_50_ values between 8 and 18 μg/mL. In general, the combination of sAuNP and natural products showed either no change (sAuNP-doxorubicin, **3**), half the activity of the natural product alone (tsitsikammamine B and 14-bromodiscorhabdin C) or significantly reduced activity (tsitsikammamine A) as shown in Table 3. Interestingly, removal of the unbound drug by centrifugation significantly reduced the biological activities of the sAuNP conjugates with IC_50_ values > 50 μg/mL. These results suggest that sAuNPs bind part of the natural product irreversibly (under the assay conditions) and prevent its interaction with its biological target. This is partly supported by the fact that removal of the ‘unbound’ portion significantly reduces the biological activity. The pH at the biological target may be an important factor ensuring the release of the natural product due to the ionic interaction between the negatively charged polysaccharide capping on the AuNP and the basic natural products. In a more acidic environment like cancerous cells, a greater payload release is expected, as shown previously by Wang et al. and Manivasagan et al. [7,26].

To determine whether the decrease in activity (Table 3) was due to poor cell penetration (as shown in Section 3.8) or poor release of the drug payload (Section 3.7), the ability of the sAuNPs to penetrate the cell membrane was carried out. The doxorubicin-bound sAuNP sample’s reduced cytotoxic activity (in comparison to **3** alone, Figure 8) is thought to be due to the poor payload release, since the conjugate is taken up by the MCF-7 cell (Figure 7) and doxorubicin alone is highly cytotoxic (Table 3). This is further supported by the release studies carried out (Table 2) where only a 17% release was achieved at neutral pHs. Manivasagan et al. loaded doxorubicin onto similarly synthesized AuNPs using Fucoidan as the capping agent [26]. It was therefore suitable to compare the sAuNPs’ performance in the doxorubicin release/desorption studies. At pH 7.4, only 17% was released from the sAuNPs compared to 10% doxorubicin from the fucoidan capped AuNPs after 72 h (Table 2). Thus, the finding was consistent, but there are still some advantages associated with a slow release of drugs in a neutral environment. At pH 4.5 however, Wang et al. and Manivasagan et al. reported a release of 97% [7,26]. Additional studies are required to investigate the effect of pH on drug release under the assay conditions. However, the uptake of the sAuNP also suggest that these conjugates could still find additional scope as multimodal agents, e.g., photothermal ablation agents in cancer therapy [26].

## 4. Conclusions

In summary, biocompatible, stable, and water soluble AuNPs (sAuNPs) were produced at room temperature and pressure conditions using an aqueous extract of the brown alga *S. incisifolium*. The aqueous extracts contained polysaccharides (Fucoidans) and polyphenols as the major and minor components, respectively, as revealed by NMR and assay data. The stability of the sAuNPs were found to be superior to that of the citrate capped AuNPs in the temperature, pH, ultracentrifugation, freeze-drying, NaCl and HSA concentration studies. The sAuNPs alone do not exert significant cytotoxic activity on their own (where ICP-OES showed that the AuNPs without a drug payload were not able to penetrate the MCF-7 cell membrane), and thus any bioactivity observed is due to the drug/metabolite payload. The known chemotherapeutic agent doxorubicin (**3**) was used as a control in the cytotoxicity studies, successfully loaded onto the sAuNP carrier and confirmed to adsorb onto the AuNP surface via the anthracene moiety, as described by Curry et al. (2015), using ^1^H NMR [32]. Doxorubicin and the pyrroloiminoquinone metabolites were successfully adsorbed onto the sAuNPs and were shown to be able to penetrate the cell membrane using ICP-OES, thus they can exert their cytotoxic activity. The activity of the pyrroloiminoquinone metabolites on their own were slightly higher than that for their nanoconjugates, however, the activity was still regarded as significant with cell viabilities at ~60–70% at 12.5 μg/mL. ICP-OES analyses revealed that the decrease in activity may be due to poor payload release rather than MCF-7 cell membrane penetration. It was also clear based on the doxorubicin observations, that the pyrroloiminoquinones are released from the sAuNP carriers/platforms since their activities is still significant. Loading the alkaloids onto a AuNP platform offers additional, more selective treatment modalities in conjunction with the bioactivity such photothermal therapy or theranostics. This work thus shows that (a) the *S. incisifolium* aqueous extract capped AuNPs are stable under physiological conditions and are biocompatible, (b) that conjugation of the sAuNPs with compounds of interest was successful, (c) that the payload is released, and (d) that the NPs bound to the compounds of interest can traverse the MCF-7 cell membrane.

## Figures and Tables

**Table 1:** Stability of sAuNPs and cAuNPs (in parentheses) under varying NaCl concentrations, human serum albumin (HSA) concentrations, temperature, and pH.

NaCl															
(mM)			SPR (nm)		Hd (nm)		HSA (mg/mL)			T (°C)			pH		
0	√	(√)	524	(524)	28.4	(10.4)	0	√	(√)	37	√	(X)	2.0	√	(X)
50	-	(√)	-	(524)	-	(28.3)	1.25	√	(X)	25	√	(√)	4.0	√	(X)
75	-	(√)	-	(524)	-	(-)	2.50	√	(X)	4	√	(√)	7.0	√	(√)
100	√	(X)	524	(703)	46.1	(811)	6.25	√	(X)	−20	√	(X)	9.0	√	(X)
1000	√	(√)	524		67.4		12.50	√	(X)	−50	√	(X)	12.0	√	(X)
5000	√	(√)	703		152.7		25.00	√	-						
6000	√	(X)	-				50.00	√	-						

cAuNPs data given in parentheses; nd: not determined; Hd: hydrodynamic diameter; X = not stable; √ = stable.

**Table 2:** Comparison of the doxorubicin-loaded sAuNPs (sAuNPs-Dox) vs. the fucoidan-capped AuNPs (Dox-Fuc-AuNPs) [26].

Parameter	sAuNPs-Dox *	Dox-Fuc-AuNPs [26]
NP reaction temperature	20 °C	80 °C
NP pH	4.1	Not reported
Payload concentration	1 mM	0.1 mM
NP conjugation method	Adjusted with NaOH ^1^	Direct
Release of payload	17% at pH 7.4 (after 72 h)	10% at pH 7.4 (after 72 h)

^1^ to facilitate the solubility of doxorubicin; * this work.

**Table 3:** IC_50_ values of compounds **3**, **5**–**7** and their sAuNP conjugates, as well as their mixtures (m) of bound and unbound AuNP conjugates, against MCF-7 cells.

	IC_50_ (μg/mL)		
Compound	Compound Alone	AuNP-1 ^a^	AuNP-2 ^b^
Doxorubicin (**3**)	17.9	15.9	>50
Tsitsikammamine A (**6**)	11.1	>50 ^c^	>50 ^c^
Tsitsikammamine B (**7**)	9.9	16.0	>50 ^c^
14-bromodiscorhabdin C (**5**)	8.3	19.9	>50 ^c^

^a^ mixture of natural product and sAuNP. ^b^ mixture of natural product and sAuNP after removal of unbound natural products. ^c^ IC_50_ > maximum treatment dose used [50 μg/mL].

## Data Availability

Not applicable.

## Supplementary Materials

The following supporting information can be downloaded at: https://www.mdpi.com/article/10.3390/ma16031319/s1, The following materials are available: Figure S1. ^1^H NMR data (DMSO-*d*_6_, 400 MHz) of Tsitsikammamine A (**6**, TA) obtained at 298 K. Figure S2. ^13^C NMR data (DMSO-*d*_6_, 100 MHz) of Tsitsikammamine A (**6**, TA) obtained at 298 K. Figure S3. ^1^H NMR data (DMSO-*d*_6_, 400 MHz) of Tsitsikammamine B (**7**, TB) obtained at 298 K. Figure S4. ^13^C NMR data (DMSO-*d*_6_, 100 MHz) of Tsitsikammamine B (**7**, TB) obtained at 298 K. Figure S5. ^1^H NMR data (DMSO-*d*_6_, 400 MHz) of 14-bromodiscorhabdin C (**5**, BDC) obtained at 298 K. Figure S6. ^13^C NMR data (DMSO-*d*_6_, 100 MHz) of 14-bromodiscorhabdin C (**5**, BDC) obtained at 298 K. Figure S7. ^1^H-^13^C HSQC NMR data (400 MHz, D_2_O) obtained from the *S. incisifolium* aqueous extract. Figure S8. 1H DOSY spectra (400 MHz) of the *S. incisifolium* aqueous extract in D_2_O at 25 °C. The values obtained in log(m^2^/s) for the polyphenol, polysaccharides and water signals are given on the spectrum. Figure S9. Typical UV/Visible spectra obtained for the AuNPs (A) synthesized using the *S. incisifolium* extract (1.0 mg and 20 mg of HAuCl_4_ salt), with absorbance spectra collected every 5 min for 4.5 h in water. As the amount of extract increased (B), the AuNPs were produced more efficiently, as revealed by the SPR band at 524 nm. Figure S10. Energy Dispersive Spectroscopy (EDS) spectra for cAuNPs (left) and sAuNPs (right). Figure S11. UV/Visible absorption spectra of the sAuNPs in a variety of NaCl concentrations. Figure S12. HRTEM images obtained for the sAuNPs following incubation with HSA. Figure S13. Reconstituted AuNPs following ultrafiltration-centrifugation. The cAuNPs lost the characteristic pink/purple color, becoming blue on conjugation, centrifugation (through the PES membrane), and freeze-drying, while the sAuNPs retained their color and morphology (please see Figure 2G, main text). Figure S14. IC_50_ value determinations for doxorubicin (**3**), 14-bromodiscorhabdin C (**5**), Tsitsikammamine A (**6**) and Tsitsikammamine B (**7**) together with the AuNP conjugates (right). Table S1: NMR data of **6** obtained in DMSO-*d*_6_ at 400 MHz at 298 K in comparison to literature values. Table S2: NMR data of **7** obtained in DMSO-*d*_6_ at 400 MHz at 298 K in comparison to literature values. Table S3: NMR spectroscopic of **5** obtained data in DMSO-*d*_6_ at 298 K in comparison to literature values.
