# Supplementary material for: Green Synthesized sAuNPs as a Potential Delivery Platform for Cytotoxic Alkaloids
Source: Materials (Basel). 2023 Feb 3;16(3):1319. doi: 10.3390/ma16031319 (PMC9920385; doi:10.3390/ma16031319)
Supplement: Supplementary file 1 [file materials-16-01319-s001.zip › materials-2113353-supplementary.pdf]

# Green synthesized sAuNPs for the delivery of cytotoxic alkaloids

Byron Mubaiwa,[a] Mookho S. Lerata,[a] Nicole R.S. Sibuyi,[b] Mervin Meyer,[b] Toufiek Samaai,[c,d] John J. Bolton,[e] Edith M. Antunes*[f] and Denzil R. Beukes*[a]

[a] School of Pharmacy, University of the Western Cape, Robert Sobukwe Road, Bellville, 7535, South Africa

[b] Department of Science and Innovation/Mintek Nanotechnology Innovation Centre (DST/Mintek NIC), Bio-labels Node, Department of Biotechnology, University of the Western Cape, Robert Sobukwe Road, Bellville, 7535, South Africa

[c] Department of Environmental Affairs (Oceans and Coasts), Cape Town, South Africa

[d] Department of Biodiversity and Conservation Biology, University of the Western Cape, Robert Sobukwe Road, Bellville, 7535, South Africa

[e] Department of Biological Sciences, University of Cape Town, Rondebosch, 7701, South Africa

[f] Department of Chemistry, University of the Western Cape, Robert Sobukwe Road, Bellville, 7535, South Africa

* Correspondence: dbeukes@uwc.ac.za; Tel.: (+27) 219592352 (DRB) and ebeukes@uwc.ac.za; Tel.: (+27) 219594020 (EMA)

# Table of Contents

1. Spectroscopic data of the pyrroloiminoquinones isolated ........ 2

2. Characterization of the *S. incisifolium* extract ........ 9

3. Synthesis and characterization of gold nanoparticles: method, results and discussion ........ 10

3.1 Citrate-capped gold nanoparticles (cAuNPs) ........ 10

3.2 *S. incisifolium* aqueous extract-capped gold nanoparticles (sAuNPs): reaction kinetics ........ 10

3.4 Characterization of the gold nanoparticles ........ 11

3.5 Stability studies of the gold nanoparticles synthesized ........ 11

3.5.1 Effect of NaCl ........ 11

3.5.2 HSA ........ 12

3.5.3 Effect of Temperature ........ 12

3.5.4 Effect of pH ........ 12

3.5.5 Effect of Freeze-drying ........ 12

3.5.6 Effect of ultracentrifugation ........ **Error! Bookmark not defined.**

# List of Figures

**Figure S1**. $^1$H NMR data (DMSO-$d_6$, 400 MHz) of Tsitsikammamine A (**6**, TA) obtained at 298K. ........ 3

**Figure S2.** $^{13}C$ NMR data (DMSO-$d_6$, 100 MHz) of Tsitsikammamine A (**6**, TA) obtained at 298K..........3

**Figure S3.** $^{1}H$ NMR data (DMSO-$d_6$, 400 MHz) of Tsitsikammamine B (**7**, TB) obtained at 298K............5

**Figure S4.** $^{13}C$ NMR data (DMSO-$d_6$, 100 MHz) of Tsitsikammamine B (**7**, TB) obtained at 298K...........5

**Figure S5.** $^{1}H$ NMR data (DMSO-$d_6$, 400 MHz) of 14-bromo discorhabdin C (**4**, BDC) obtained at 298K. ...............7

**Figure S6.** $^{13}C$ NMR data (DMSO-$d_6$, 100 MHz) of 14-bromo discorhabdin C (**4**, BDC) obtained at 298K. ...............7

**Figure S7.** $^{1}H$-$^{13}C$ HSQC NMR data (400 MHz, $D_2O$) obtained from the *S. incisifolium* aqueous extract.9

**Figure S8.** $^{1}H$ DOSY spectra (400 MHz) of the *S. incisifolium* aqueous extract in $D_2O$ at 25 °C. The values obtained in log($m^2$/s) for the polyphenol, polysaccharides and water signals are given on the spectrum ..........7

**Figure S9.** Typical UV/Visible spectra obtained for the AuNPs (A) synthesized using the *S. incisifolium* extract (1.0 mg and 20 mg of $HAuCl_4$ salt), with absorbance spectra collected every 5 minutes for 4.5 hrs in water. As the amount of extract increased (B), the AuNPs were produced more efficiently as revealed by the SPR band at 524 nm. ..........10

**Figure S10.** Energy Dispersive Spectroscopy (EDS) spectra for cAuNPs (left) and sAuNPs (right). ....11

**Figure S11.** UV/Visible absorption spectra of the sAuNPs in a variety of NaCl concentrations. ..........11

**Figure S12.** HRTEM images obtained for the sAuNPs following incubation with HSA. .......................12

**Figure S13.** Reconstituted AuNPs following ultrafiltration-centrifugation. The cAuNPs lost the characteristic pink/purple colour becoming blue on conjugation, centrifugation (through the PES membrane), and freeze-drying, while the sAuNPs retained their colour and morphology (please see Figure 2G, main text). ..........13

**Figure S14.** $IC_{50}$ value determinations for doxorubicin (**3**), 14-bromodiscorhabdin C (**5**), Tsitsikammamine A (**6**) and Tsitsikammamine B (**7**) together with the AuNP conjugates (right)……14

## List of Tables

**Table S1:** NMR data of **6** obtained in DMSO-$d_6$ at 400 MHz at 298K in comparison to literature values. ..........4

**Table S2:** NMR data of **7** obtained in DMSO-$d_6$ at 400 MHz at 298K in comparison to literature values. ..........6

**Table S3:** NMR spectroscopic of **4** obtained data in DMSO-$d_6$ at 298K in comparison to literature values. ..........8

## 1. Spectroscopic data of the pyrroloiminoquinones isolated

**Tsitsikammamine A (6):** a reddish-brown solid (yield: 9.0 mg), characterized as its TFA salt. LRESIMS *m/z* 304.70 [M]$^+$ (calc. for $C_{18}H_{14}N_3O_2$, 304.12).

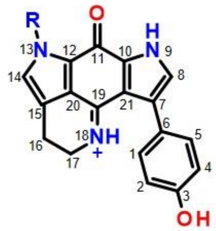


R = H

**6, TA**

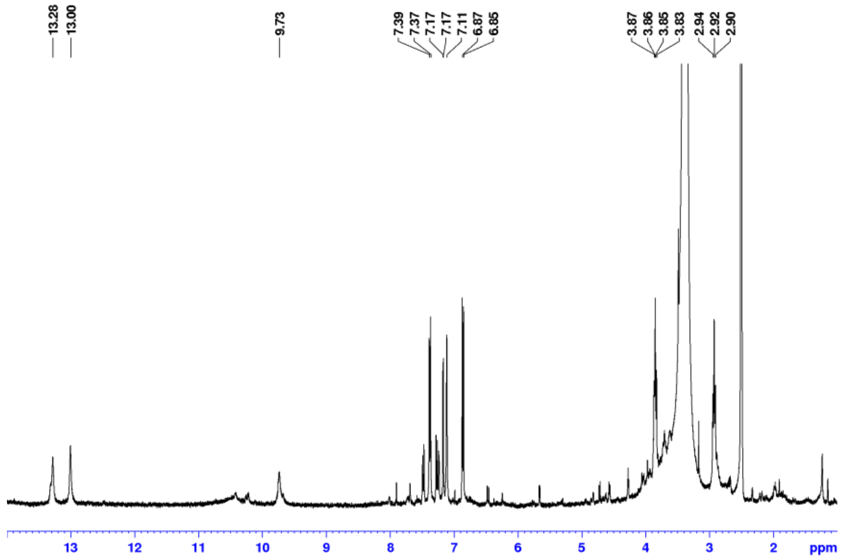


**Figure S1**. $^1$H NMR data (DMSO-$d_6$, 400 MHz) of Tsitsikammamine A (**6**, TA) obtained at 298K.

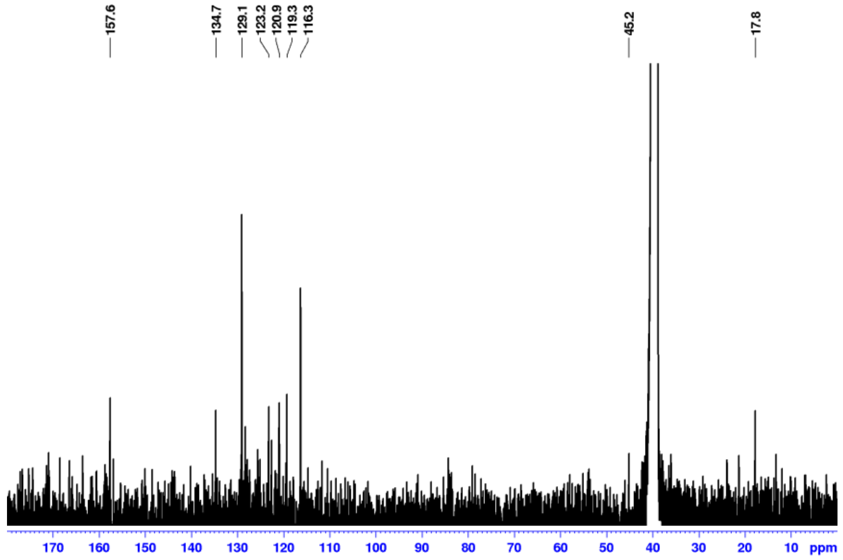


**Figure S2.** $^{13}$C NMR data (DMSO-$d_6$, 100 MHz) of Tsitsikammamine A (**6**, TA) obtained at 298K.

**Table S1:** NMR data of **6** obtained in DMSO-$d_6$ at 400 MHz at 298K in comparison to literature values.

| **Atom** | **δ$_C$ (multi.)** (this work) | **δ$_H$ (int., multi., *J* in Hz)** (this work) | **δ$_H$ (int., multi., *J* in Hz)** **[1,2]** | **δ$_C$ (multi.)** **[1,2]** |
|---|---|---|---|---|
| 1 | 116.3 (CH) | 6.86 (2H, *d*, 8.5 Hz) | 6.87 (*d*, 8.4 Hz) | 116.2 ($CH_2$) |
| 2 | 129.1 (CH) | 7.38 (2H, *d*, 8.4 Hz) | 7.37 (*d*, 8.4 Hz) | 128.9 ($CH_2$) |
| 3 | 157.6 (C) | — | — | 157.6 (C) |
| 4 | 129.1 (CH) | 7.38 (2H, *d*, 8.4 Hz) | 7.37 (*d*, 8.4 Hz) | 128.9 (C) |
| 5 | 116.3 (CH) | 6.86 (2H, *d*, 8.5 Hz) | 6.87 (*d*, 8.4 Hz) | 116.2 ($CH_2$) |
| 6 | 127.2 (C)* | — | — | 127.2 (C) |
| 7 | 122.4 (C)* | — | — | 122.4 (C) |
| 8 | 125.6 (CH) | 7.17 (1H, *d*, 2.8 Hz) | 7.16 (*d*, 2.5 Hz) | 125.0 ($CH_2$) |
| 10 | 134.7 (C) | — | — | 134.6 (C) |
| 11 | 166.8 (C) | — | — | 166.3 (C) |
| 12 | 126.4 (C)* | — | — | 127.8 (C) |
| 14 | 123.6 (C) | 7.11 (1H, *s*) | 7.10 (*d*, 1.8 Hz) | 123.1 (CH) |
| 15 | 119.3 (C)* | — | — | 119.2 (C) |
| 16 | 17.8 ($CH_2$) | 2.92 (2H, *t*, 7.8 Hz) | 2.93 (*t*, 7.8 Hz) | 17.6 ($CH_2$) |
| 17 | 45.2 ($CH_2$) | 3.85 (2H, *t*, 7.8 Hz) | 3.85 (*t*, 7.8 Hz) | 45.0 ($CH_2$) |
| 19 | 156.2 (C)* | — | — | 157.6 (C) |
| 20 | 113.7 (C)* | — | — | 113.5 (C) |
| 21 | 121.0 (C)* | — | — | 120.7 (C) |
| *N*9 | — | 13.28 (1H, br. *s*) | 13.28 (br. *s*) | — |
| *N*13 | — | 13.00 (1H, s) | 13.01 (br. *s*) | — |
| *N*18 | — | 9.75 (1H, br. *s*) | 9.75 (br. *s*) | — |

* Chemical shifts obtained from 2D data.

**Tsitsikammamine B (7):** dark red solid (yield: 23.2 mg), characterized as its TFA salt. HRESIMS *m/z* 318.1237 [M]$^+$ (calc. for $C_{19}H_{16}N_3O_2$, 318.1321).

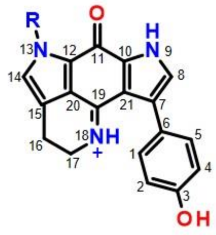


R = $CH_3$
**7, TB**

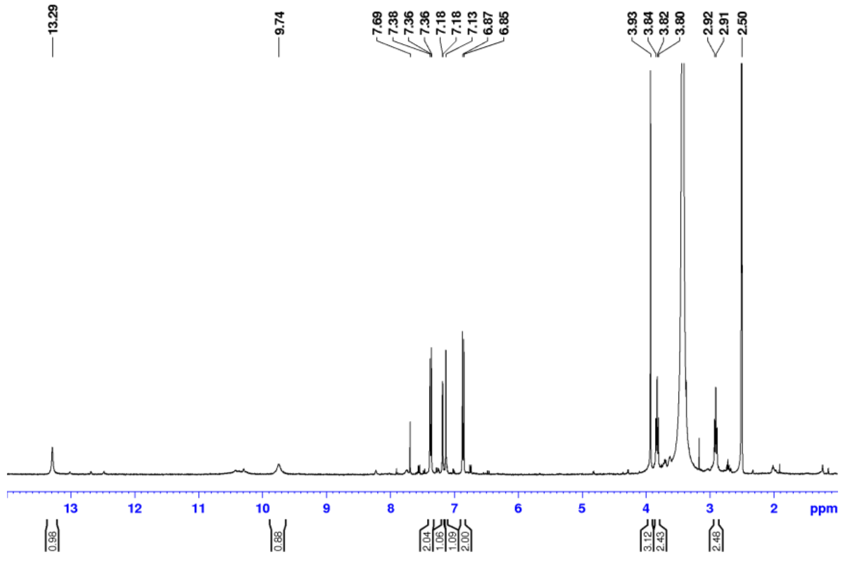


**Figure S3.** $^1$H NMR data (DMSO-$d_6$, 400 MHz) of Tsitsikammamine B (**7**, TB) obtained at 298K.

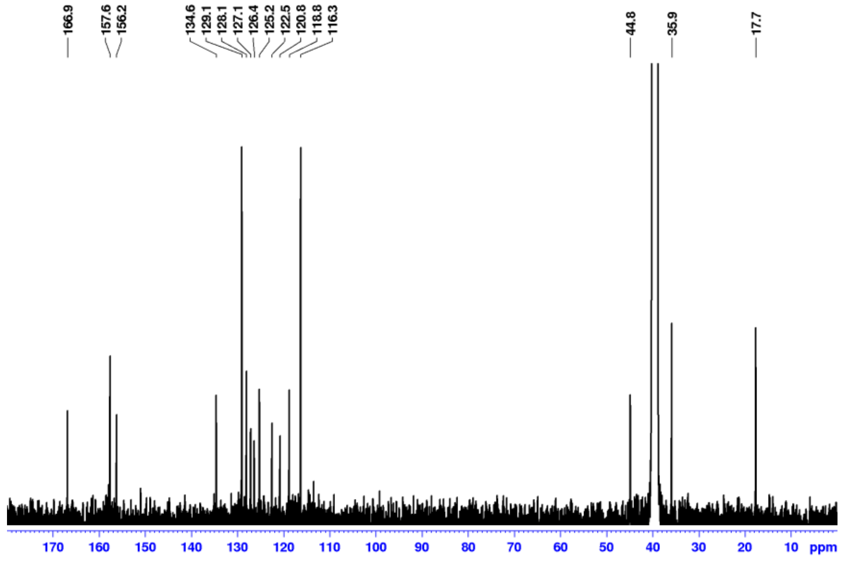


**Figure S4.** $^{13}$C NMR data (DMSO-$d_6$, 100 MHz) of Tsitsikammamine B (**7**, TB) obtained at 298K.

**Table S2:** NMR data of **7** obtained in DMSO-$d_6$ at 400 MHz at 298K in comparison to literature values.

| Atom | **δ$_C$ (multi.)** (this work) | **δ$_H$ (int., multi., *J* in Hz)** (this work) | **δ$_H$ (int., multi., *J* in Hz)** **[1,2]** | **δ$_C$ (multi.)** **[1,2]** |
|---|---|---|---|---|
| 1 | 116.3 (CH) | 6.86 (2H, *d*, 8.2 Hz) | 6.86 (*d*, 8.1 Hz) | 116.3 ($CH_2$) |
| 2 | 129.1 (CH) | 7.37 (2H, *d*, 8.6 Hz) | 7.36 (d, 8.1 Hz) | 129.0 ($CH_2$) |
| 3 | 157.6 (C) | — | — | 157.6 (C) |
| 4 | 129.1 (CH) | 7.37 (2H, *d*, 8.2 Hz) | 7.36 (*d*, 8.1 Hz) | 129.0 (CH) |
| 5 | 116.3 (CH) | 6.86 (2H, *d*, 8.2 Hz) | 6.86 (*d*, 8.1 Hz) | 116.3 ($CH_2$) |
| 6 | 127.2 (C) | — | — | 127.1 (C) |
| 7 | 122.4 (C) | — | — | 122.4 (C) |
| 8 | 125.3 (CH) | 7.18 (1H, *d*, 3.1 Hz) | 7.16 (br. *s*) | 125.2 ($CH_2$) |
| 10 | 134.6 (C) | — | — | 134.6 (C) |
| 11 | 166.8 (C) | — | — | 166.8 (C) |
| 12 | 126.4 (C) | — | — | 126.4 (C) |
| 14 | 128.1 (C) | 7.13 (1H, *s*) | 7.11 (*s*) | 128.0 (C) |
| 15 | 118.8 (C) | — | — | 118.7 (C) |
| 16 | 17.7 ($CH_2$) | 2.91 (2H, *t*, 8.0) | 2.91 (*t*, 7.8 Hz) | 17.6 ($CH_2$) |
| 17 | 44.8 ($CH_2$) | 3.82 (2H, *t*, 8.0) | 3.82 (*t*, 7.8 Hz) | 44.7 ($CH_2$) |
| 19 | 156.2 (C) | — | — | 156.2 (C) |
| 20 | 112.9 (C) | — | — | 113.3 (C) |
| 21 | 120.8 (C) | — | — | 120.7 (C) |
| *NH*9 | — | 13.29 (1H, br. *s*) | 13.3 (br. *s*) | — |
| *NH*18 | — | 9.75 (1H, br. *s*) | 9.80 (br. *s*) | — |
| *N*13-$CH_3$ | 35.9 ($CH_3$) | 3.92 (3H, s) | 3.92 (s) | 35.8 ($CH_3$) |

**14-Bromo-discorhabdin C (5):** a reddish-brown solid (yield: 15.5 mg), characterized as its TFA salt. HRESIBMS *m/z* $[M]^+$ 539.8521 (calcd for $C_{18}H_{13}N_3O_2{}^{79}Br_3$, 539.8558).

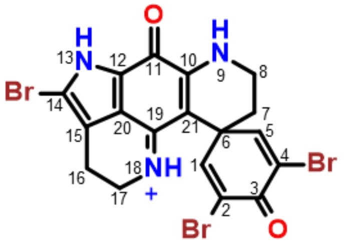

**5, BCD**

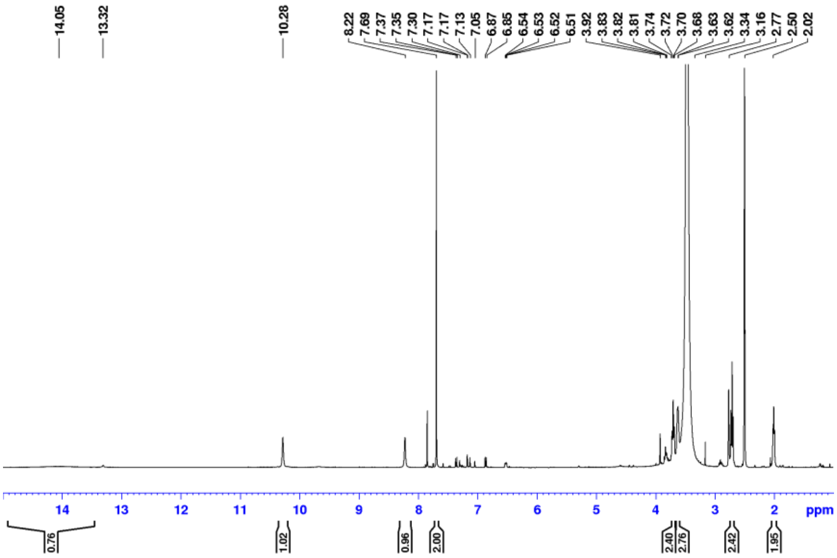


**Figure S5.** $^1H$ NMR data (DMSO-$d_6$, 400 MHz) of 14-bromo discorhabdin C (**5**, BDC) obtained at 298K.

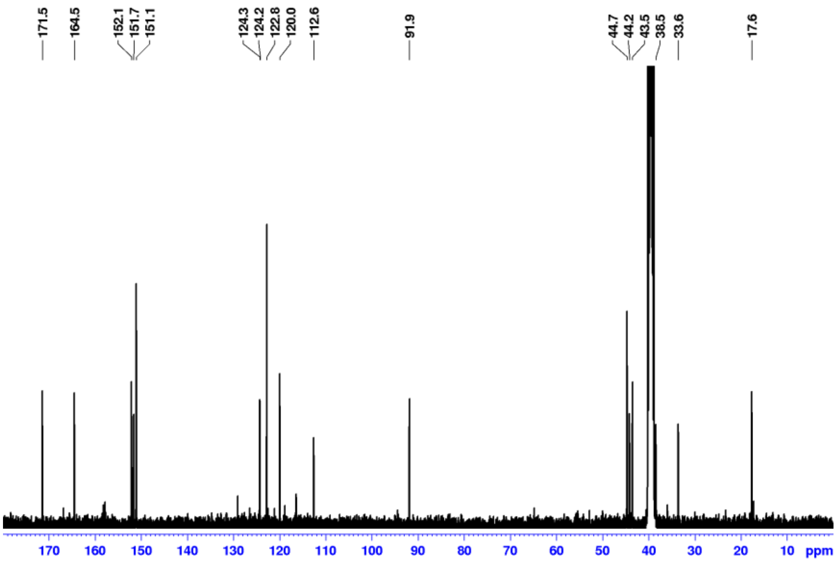


**Figure S6.** $^{13}C$ NMR data (DMSO-$d_6$, 100 MHz) of 14-bromo discorhabdin C (**5**, BDC) obtained at 298K.

**Table S3:** NMR spectroscopic of **5** obtained data in DMSO-$d_6$ at 298K in comparison to literature values.

| **Atom** | **δ$_C$ (mult.)** (this work) | **δ$_H$ (int., multi., *J* in Hz)** (this work) | **δ$_H$ (int., multi., *J* in Hz)** **[1,2]** | **δ$_C$ (multi.)** **[1,2]** |
|---|---|---|---|---|
| 1 | 151.1 (CH) | 7.69 (2H, *s*) | 7.70 (*s*) | 150.9 (CH) |
| 2 | 122.8 (C) | — | — | 122.6 (C) |
| 3 | 171.5 (C) | — | — | 171.2 (C) |
| 4 | 122.8 (C) | — | — | 122.6 (C) |
| 5 | 151.1 (CH) | 7.69 (2H, *s*) | 7.70 (*s*) | 150.9 (CH) |
| 6 | 44.7 (C) | — | — | 44.5 (C) |
| 7 | 33.6 (CH) | 2.01 (2H, *t*, 5.6) | 2.02 (br. *s*) | 33.5 ($CH_2$) |
| 8 | 38.5 (CH) | 3.63 (2H, *m*) | 3.63 (br. *s*) | 38.3 ($CH_2$) |
| 10 | 151.7 (C) | — | — | 151.5 (C) |
| 11 | 164.5 (C) | — | — | 164.3 (C) |
| 12 | 124.2 (C) | — | — | 124.1 (C) |
| 14 | 112.6 (C) | — | — | 112.5 (C) |
| 15 | 120.0 (C) | — | — | 119.8 (C) |
| 16 | 17.6 ($CH_2$) | 2.71 (2H, *t*, 7.4 Hz) | 2.71 (*t*, 7.4 Hz) | 17.5 ($CH_2$) |
| 17 | 43.5 ($CH_2$) | 3.71 (2H, *t*, 7.4 Hz) | 3.71 (*t*, 7.4 Hz) | 43.4 ($CH_2$) |
| 19 | 152.1 (C) | — | — | 152.0 (C) |
| 20 | 91.9 (C) | — | — | 91.7 (C) |
| 21 | 124.3 (C) | — | — | 124.2 (C) |
| *NH*9 | — | 10.29 (1H, br. *s*) | 10.27 (br. *s*) | — |
| *NH*13 | — | 14.10 (1H, br. *s*) | 13.0 (br. *s*) | — |
| *NH*18 | — | 8.23 (1H, br. *s*) | 8.22 (br. *s*) | — |

## 2. Characterization of the *S. incisifolium* extract

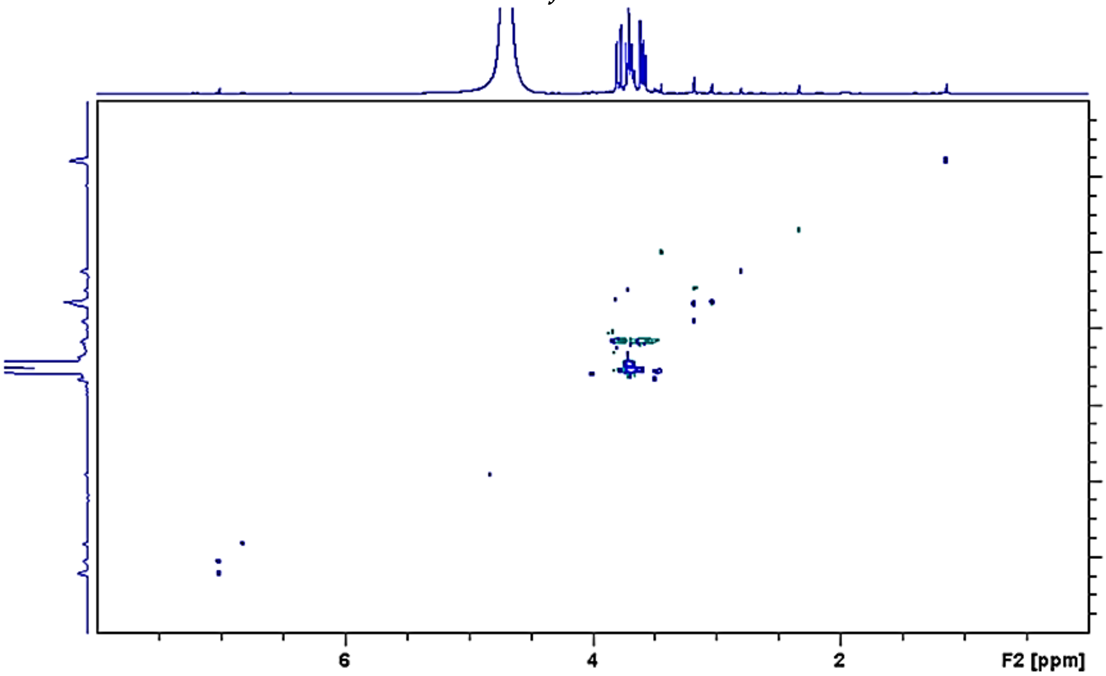


**Figure S7.** $^{1}H$-$^{13}C$ HSQC NMR data (400 MHz, $D_2O$) obtained from the *S. incisifolium* aqueous extract.

Figure S7 shows the HSQC data, which is useful in determining the major components of the *S. incisifolium* aqueous extract. Overlapping $^{1}H$ signals are dispersed in a second dimension, which is not possible with 1D $^{1}H$ and $^{13}C$ NMR data.

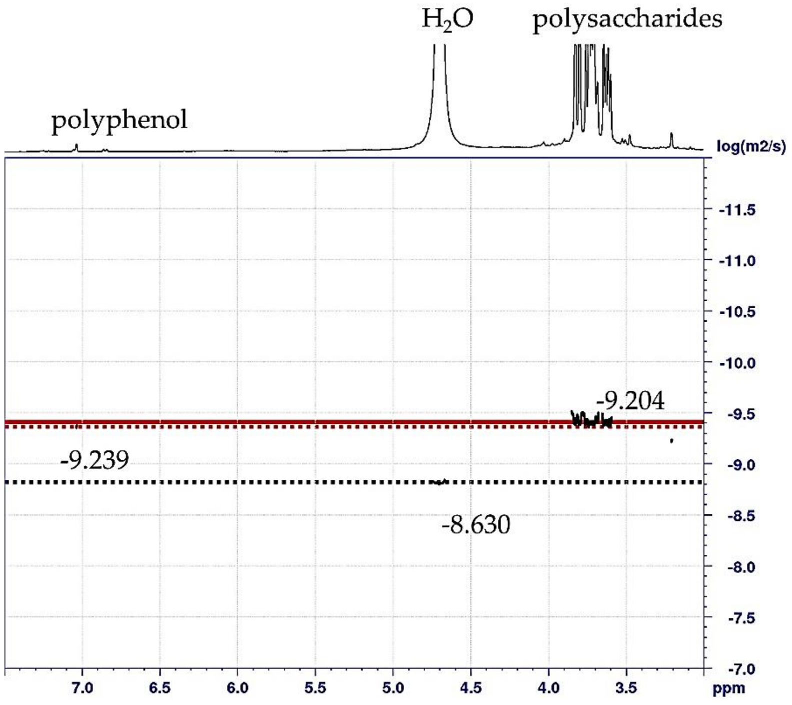


**Figure S8.** $^{1}H$ DOSY spectra (400 MHz) of the *S. incisifolium* aqueous extract in $D_2O$ at 25 °C. The values obtained in log($m^2$/s) for the polyphenol, polysaccharides and water signals are given on the spectrum.

## 3. Synthesis and characterization of gold nanoparticles: method, results, and discussion

### 3.1 Citrate-capped gold nanoparticles (cAuNPs)

The method used to prepare these nanoparticles is previously described by Turkevich, et al. (1951). Briefly, 30 mL of a 10.015 mM of sodium citrate dihydrate solution was transferred to a round bottom flask and approximately 117.5 mL of water was added to the flask and brought to boil (without stirring) at which point 2.5 mL of a 10.162 mM $HAuCl_4$ solution was added.

### 3.2 *S. incisifolium* aqueous extract-capped gold nanoparticles (sAuNPs): reaction kinetics

To a 0.15 mM solution of gold salt (1 mL in 10 mL), 1.0, 2.0 and 3.75 mg of the *Sargassum* extract was added and stirred. 500-μL aliquots were and made up to 3 mL and analyzed by UV-Visible spectroscopy (Figure S9) every 5 minutes for 4.5 hours.

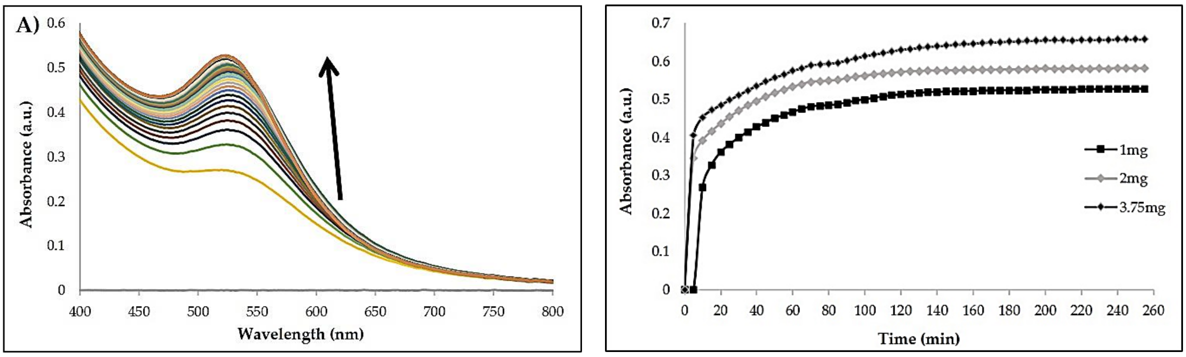


**Figure S9.** Typical UV/Visible spectra obtained for the AuNPs (A) synthesized using the *S. incisifolium* extract (1.0 mg and 20 mg of HAuCl4 salt), with absorbance spectra collected every 5 minutes for 4.5 hrs in water. As the amount of extract increased (B), the AuNPs were produced more efficiently as revealed by the SPR band at 524 nm.

### 3.3 Optimization of extract-capped nanoparticles

Several protocols were attempted in the overall synthetic processes using the *Sargassum* seaweed extract. The extract used is as described in the main paper. Firstly, 20 mg of the gold salt solution (in 100 mL) was added to 2.5 mg of the extract (s) and the mixture allowed to stir at room temperature (A) for 1 hour. Process A was repeated except 10 mg of the extract was used and the reaction mixture irradiated using a Halogen (365 nm, 45W) UV-lamp (B). Process C followed that of B, except 25 mg of the extract was used instead. Finally, the amount of extract was increased to 120 mg (D). Process D was deemed to be the most suitable and eventually used for the preparation of the nanoparticles used to load the doxorubicin and pyrroloiminoquinone metabolites.

### 3.4 Characterization of the gold nanoparticles

EDS spectra obtained for the cAuNPs and sAuNPs are shown in Figure S10, and reveal the presence of Au, as expected. However, the spectra obtained for the cAuNPs were more clearly defined.

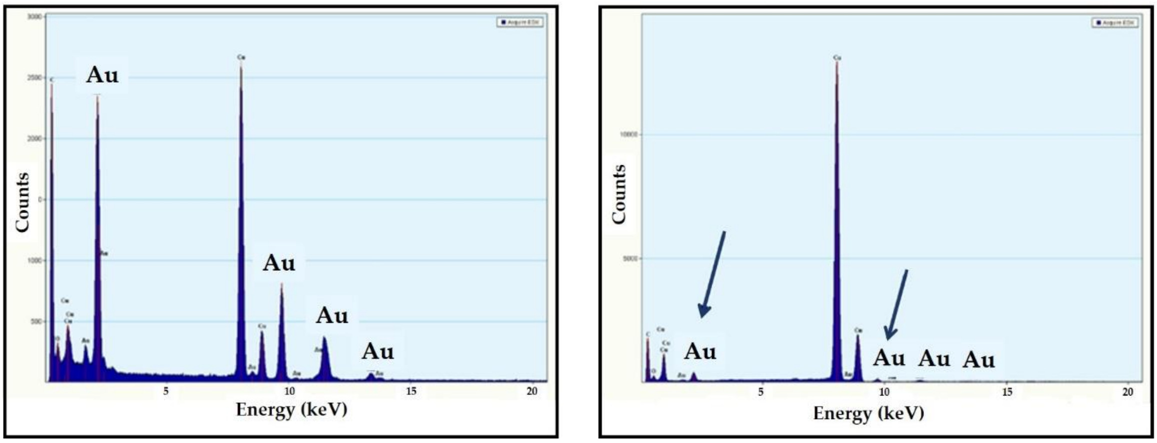


**Figure S10.** Energy Dispersive Spectroscopy (EDS) spectra for cAuNPs (left) and sAuNPs (right).

### 3.5 Stability studies of the gold nanoparticles synthesized

All measurements were easily carried using a UV-Visible spectrometer using a scanning range of 800 nm to 200 nm. The reaction process was acquired using the 'cycle mode' on a Cary UV-Visible Spectrometer, with a scan set to acquire data every 2.5 minutes or as previously described. The plots are shown in Figure S9.

#### 3.5.1 Effect of NaCl

To 500 μL of the s- or c-AuNPs, 2.5 mL of a 100 mM, 1000 mM, 5000 mM, and 6000 mM of a NaCl solution was added and the sample measured using UV-Visible spectroscopy (Figure S11), the zeta-sizer and zeta potential (DLS). The cAuNPs were prepared according to the Turkevich approach [3]. For the long-term study, 1 mL of the s- or c-AuNPs solution was mixed with 5 mL of each of the respective concentrations of NaCl.

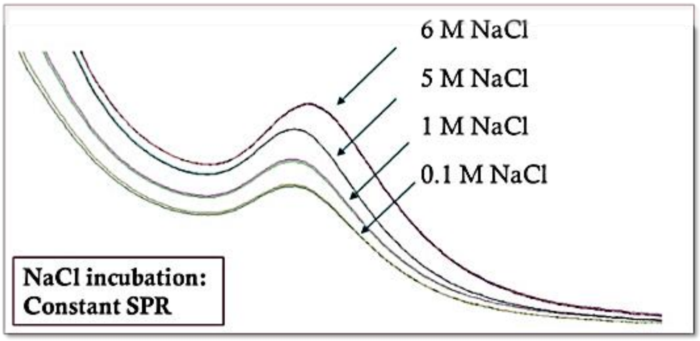


**Figure S11.** UV/Visible absorption spectra of the sAuNPs in a variety of NaCl concentrations.

### 3.5.2 HSA

1 mL of sAuNPs or cAuNPs were used for the HSA experiments at concentrations of 1.25, 2.50, 6.25, 12.5, 25.0 and 50.0 mg/mL in MilliQ water. The HSA sample was analyzed using HRTEM (please see Figure S12) in addition to UV/Visible spectroscopy.

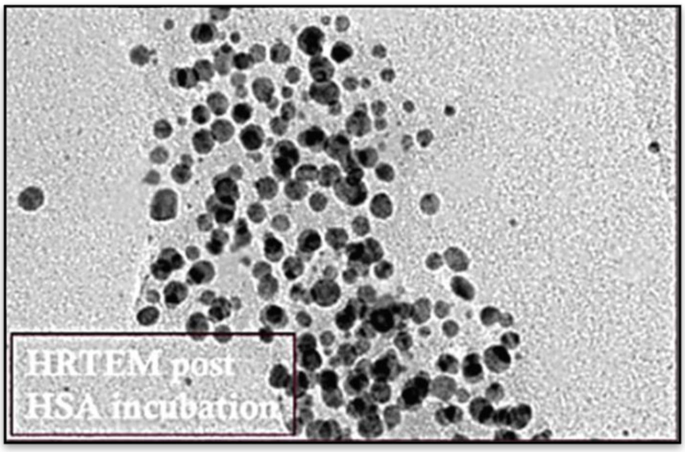


**Figure S12.** HRTEM images obtained for the sAuNPs following incubation with HSA.

### 3.5.3 Effect of Temperature

3 mL of sAuNPs or cAuNPs was used for the study at each of the temperatures at -50, -20, 4, 25 and 37 $^{o}$C. 500 μL of the NP solution was sampled for UV-Visible measurements for four weeks. These samples were used for the DLS measurements.

### 3.5.4 Effect of pH

1 mL of the sAuNPs or cAuNPs was used at each of the pH conditions at 2.0, 4.0, 7.0, 9.0 and 12.0. 1 mL was then sampled every week to assess the stability using UV/Visible spectroscopy and DLS measurements.

### 3.5.5 Effect of Freeze-drying and ultracentrifugation

The stability of the sAuNPs against freeze-drying was also assessed where the NPs were subjected to 5 freeze-drying (thaw) cycles. The samples maintained their solubility and structural integrity. The samples sAuNPs-5 and sAuNPs-40, where 5 mg and 40 mg of extract were used to produce the nanoparticles respectively, were shell-frozen using liquid nitrogen and allowed to freeze dry overnight, for five cycles. A sample of cAuNPs was used as a control. Following each drying cycle, the powdered nanoparticles were reconstituted in water and the UV-Visible absorption spectra acquired.

Colloidal suspensions of the gold nanoparticles (sAuNPs or cAuNPs) were, at random intervals, placed into a 2 mL centrifugal concentrator and centrifuged (Figure S13). The

particles trapped in the collector were reconstituted in water, recovered and the UV-Visible absorption spectra acquired. Processes such as ultrafiltration and centrifugation processes were challenging for the cAuNPs (the samples were challenging to reconstitute, and the colour changed to blue) but not so for the sAuNPs. This step was necessary to assess since conjugation of the AuNPs with metabolites necessitates the determination of binding efficiency. The cAuNPs were not able to withstand centrifugation and ultrafiltration using the Vivaspin® centrifugal concentrators (Figure S13). The sAuNPs were easily reconstituted, maintaining the SPR band wavelength and colour.

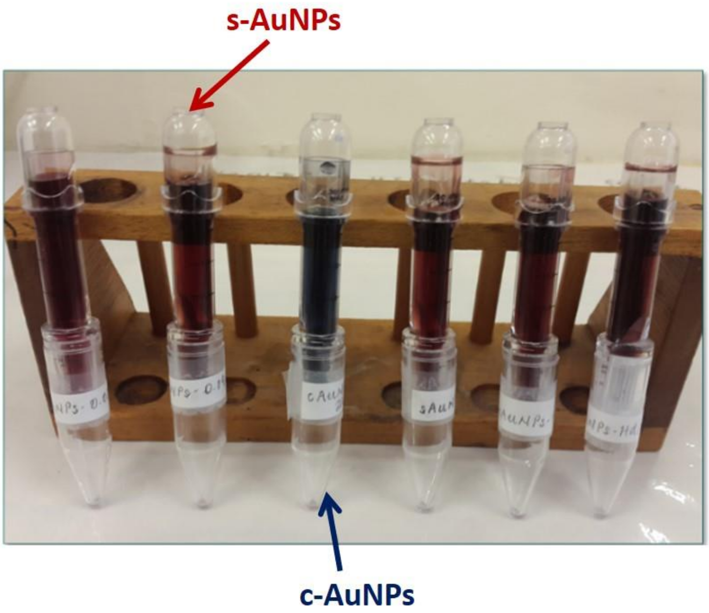


**Figure S13.** Reconstituted AuNPs following ultrafiltration-centrifugation. The cAuNPs lost the characteristic pink/purple colour becoming blue on conjugation, centrifugation (through the PES membrane), and freeze-drying, while the sAuNPs retained their colour and morphology (please see Figure 3G, main text).

### 3.6 Biological studies

The $IC_{50}$ values were determined for the compounds alone as well as their NP conjugates (Figure S14).

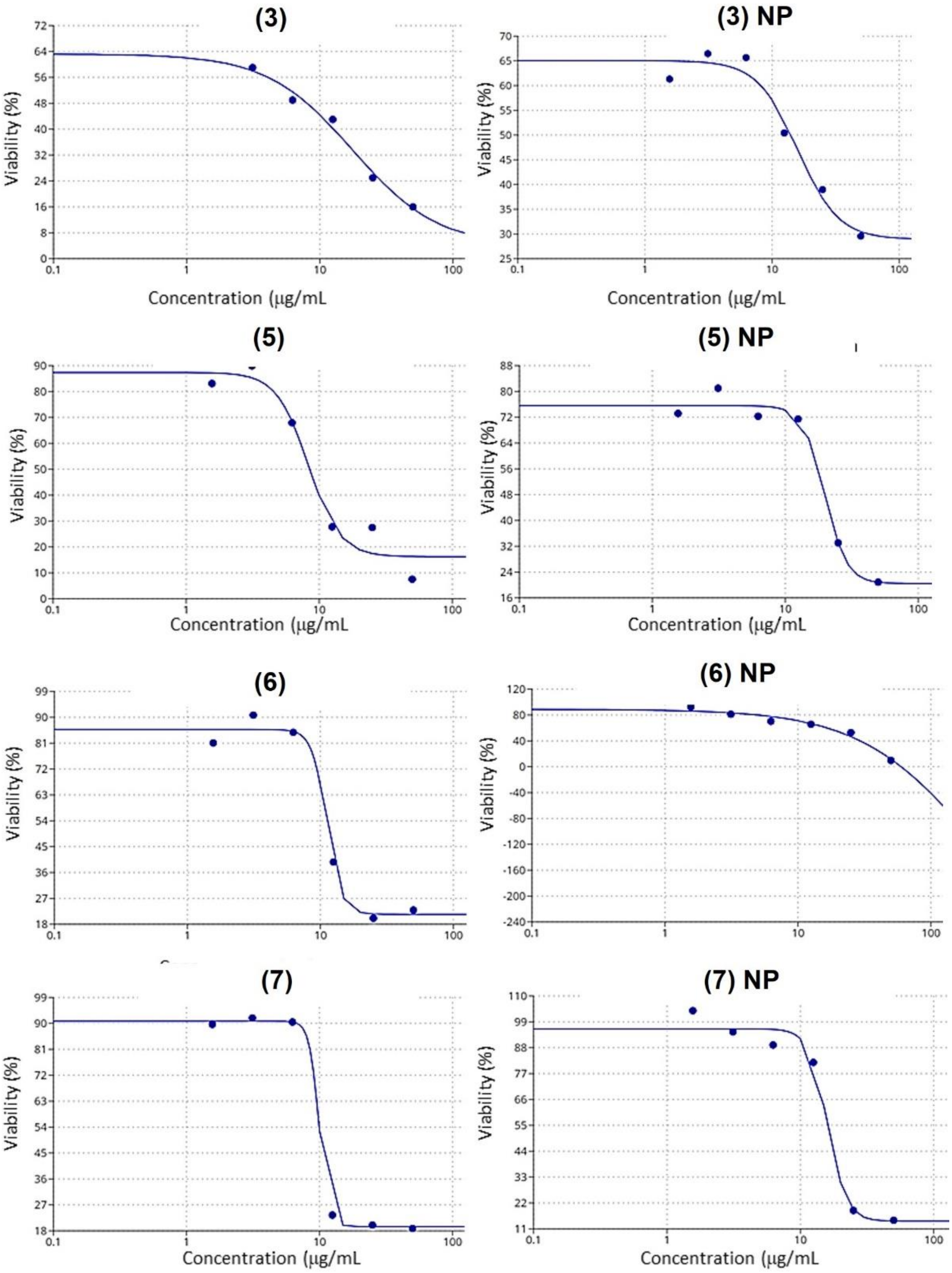


***Figure S14.*** *IC*$_{50}$ *value determinations for doxorubicin (3), 14-bromodiscorhabdin C (5), Tsitsikammamine A (6) and Tsitsikammamine B (7) together with the AuNP conjugates (right).*

**References:**


1. Antunes, E.M.; Beukes, D.R.; Kelly, M.; Samaai, T.; Barrows, L.R.; Marshall, K.M.; Sincich, C.; Davies-Coleman, M.T. *J. Nat. Prod.* **2004**, *67*(8), 1268-1276.
2. Hooper, G.; Davies-Coleman, M.; Kelly-Borges, M.; Coetzee, P. *Tetrahed Lett.* **1996**, *37*(39), 7135-7138.
3. Turkevich, J.; Stevenson, P.; Hillier, J. *Discuss. Faraday Soc.* **1951**, *11*, 55-75.
